# Cd^2+^-Selective Fluorescence Enhancement of Bisquinoline Derivatives with 2-Aminoethanol Skeleton

**DOI:** 10.3390/molecules29020369

**Published:** 2024-01-11

**Authors:** Yuji Mikata, Aya Tsuruta, Hinata Koike, Sunao Shoji, Hideo Konno

**Affiliations:** 1 Laboratory for Molecular & Functional Design, Department of Engineering, Nara Women’s University, Nara 630-8506, Japan; 2 KYOUSEI Science Center, Nara Women’s University, Nara 630-8506, Japan; 3 Department of Chemistry, Biology and Environmental Science, Faculty of Science, Nara Women’s University, Nara 630-8506, Japan; 4 Cooperative Major in Human Centered Engineering, Nara Women’s University, Nara 630-8506, Japan; 5 National Institute of Advanced Industrial Science and Technology (AIST), 1-1-1 Higashi, Tsukuba 305-8565, Japan

**Keywords:** cadmium, fluorescence, sensor, zinc, quinoline, 2-aminoethanol, ligand effect

## Abstract

The development of fluorescent Cd^2+^ sensors requires strict selectivity over Zn^2+^ because of the high availability of Zn^2+^ in the natural environment. In this paper, bisquinoline-based fluorescent sensors with a 2-aminoethanol backbone were investigated. The weak coordination ability of quinoline compared to well-studied pyridine is suitable for Cd^2+^ selectivity rather than Zn^2+^. In the presence of 3 equiv. of metal ions, TriMeO-*N,O*-BQMAE (*N,O*-bis(5,6,7-trimethoxy-2-quinolylmethyl)-2-methylaminoethanol (**3**)), as well as its *N,N*-isomer TriMeO-*N,N*-BQMAE (*N,N*-bis(5,6,7-trimethoxy-2-quinolylmethyl)-2-methoxyethylamine (**6**)), exhibits Cd^2+^-selective fluorescence enhancement over Zn^2+^ in DMF-HEPES buffer (1:1, 50 mM HEPES, 0.1 M KCl, pH = 7.5) (*I*_Zn_/*I*_Cd_ = 26–34%), which has similar selectivity in comparison to the corresponding ethylenediamine derivative TriMeOBQDMEN (*N,N’*-bis(5,6,7-trimethoxy-2-quinolylmethyl)-*N,N’*-dimethylethylenediamine) under the same experimental condition (*I*_Zn_/*I*_Cd_ = 24%). The fluorescence mechanisms of *N,O*- and *N,N*-isomers of BQMAE are quite different, judging from the fluorescence lifetimes of their metal complexes. The Cd^2+^ complex with TriMeO-*N,O*-BQMAE (**3**) exhibits a long fluorescence lifetime similar to that of TriMeOBQDMEN via intramolecular excimer emission, whereas the Cd^2+^ complex with TriMeO-*N,N*-BQMAE (**6**) exhibits a short lifetime from monomer emission.

## 1. Introduction

Cadmium is a toxic metal and exists in vast areas of natural environments. Extensive use of cadmium in industry, including batteries and pigments, facilitates the exposure of cadmium to the air, water, and soils. As a result, cadmium-related health problems in humans are recent serious concerns [1,2]. In this context, the detection and quantification of cadmium in the environment, especially in water, are of urgent demand [3,4,5].

The fluorescence method is a powerful tool for the detection of metal ions and biologically important small molecules [6,7,8,9,10,11,12,13,14,15]. Its sensitivity, rapidness, and convenience allow us to visualize the target effectively. Specificity is the most important requisite for fluorescence sensing and owes to the rational molecular design of fluorescent probes. One of the most difficult and important problems in fluorescence detection is the discrimination between Cd^2+^ and Zn^2+^ because these two metal ions slightly differ in their ionic radii, which is only 21 pm [16,17,18,19]. The weak basicity of the quinoline nitrogen atom compared to that of pyridine offers a suitable coordination environment around the larger and softer Cd^2+^ center than Zn^2+^. Therefore, many quinoline-based fluorescent Cd^2+^ sensors have been extensively developed [20,21,22,23,24,25].

**Scheme 1:**
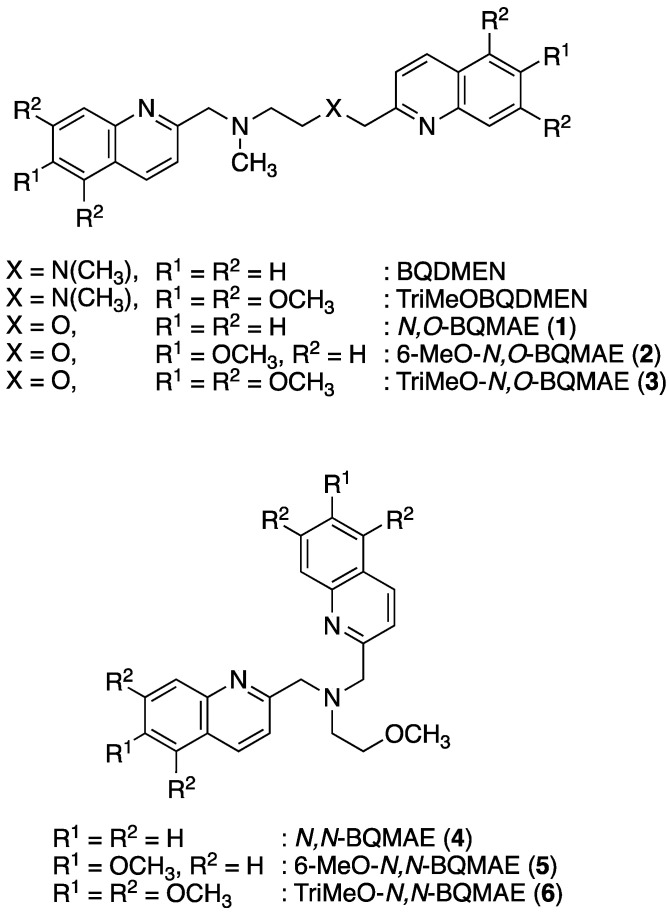
Structure of ligands.

In our laboratory, several fluorescent probes for Zn^2+^ and Cd^2+^ based on the polyquinoline structure have been reported [26]. Considering the larger coordination number of Cd^2+^ in comparison to Zn^2+^, the hepta- and octadentate tetrakisquinoline derivatives such as TQOPEN [27] (*N,N,N’,N’*-tetrakis(2-quinolylmethyl)-3-oxa-1,5-pentanediamine) and TriMeOBAPTQ [28] (*N,N,N’,N’*-tetrakis(5,6,7-trimethoxy-2-quinolylmethyl)-2,2′-(*N,N’*-dimethylethylenediamino)dianiline) were developed. Recently, the tetradentate bisquinoline derivative TriMeOBQDMEN [29] (*N,N’*-bis(5,6,7-trimethoxy-2-quinolylmethyl)-*N,N’*-dimethylethylenediamine, Scheme 1) opened the door to a new field of easily accessible small molecular fluorescent sensors for Cd^2+^. The TriMeOBQDMEN exhibits Cd^2+^-selective fluorescence enhancement in DMF-H_2_O (1:1) (*I*_Zn_/*I*_Cd_ = 22% in the presence of 1 equiv. of metal ion). The fluorescent Cd^2+^/Zn^2+^ discrimination mechanism of TriMeOBQDMEN is not fully understood; however, the bis(µ-chloro) dicadmium complex was characterized using BQDMEN (Scheme 1) as a plausible fluorescent species [29]. In order to further develop the potential of tetradentate bisquinoline derivatives as Cd^2+^-specific fluorescent sensors, here we designed oxygen-containing N3O1 tetradentate bisquinoline derivatives of BQDMEN, namely, *N,O*-BQMAE (*N,O*-bis(2-quinolylmethyl)-2-methylaminoethanol (**1**)) and its methoxy-substituted analogs **2** and **3** (Scheme 1). These oxygen-containing ligands were expected to possess reduced metal binding affinity compared with N4 tetradentate ligands and, therefore, a library with a set of fluorescent ligands with a wide variety of thresholds to quantify the concentration of Cd^2+^ can be constructed. Moreover, *N,N*-BQMAE (*N,N*-bis(2-quinolylmethyl)-2-methoxyethylamine (**4**)) and its methoxy-substituted analogs **5** and **6** (Scheme 1) were also examined as a set of regioisomers of *N,O*-BQMAE derivatives to expand the compound library.

## 2. Results and Discussion

### 2.1. Synthesis of Ligands

The newly designed six ligands (**1**–**6**) based on a 2-aminoethanol skeleton were prepared according to Scheme S1 using corresponding amines and methoxy-substituted chloromethylquinolines. N-alkylation of 2-methylaminoethanol followed by O-alkylation using *n*-butyllithium affords **1**–**3** in quite low yields (6–19%) due to the unoptimized experimental procedure for the second step. The synthesis of **4**–**6** was conducted in one step, starting from 2-methoxyethylamine in excellent yields (96% to quantitative yield). Structure and purity of ligands **3**–**6** were supported by ^1^H/^13^C NMR and elemental analysis. Structure of ligands **1** and **2** was confirmed by ^1^H/^13^C NMR. The complicated coupling pattern at the aliphatic proton region of **2** in ^1^H NMR spectrum is still under investigation.

### 2.2. Fluorescence Spectral Changes of N,O-BQMAE Derivatives ***1**–**3***

**Figure 1:**
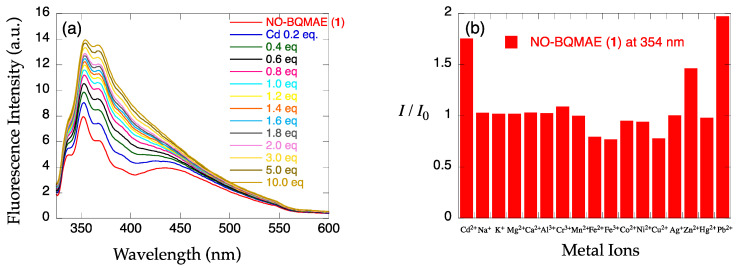

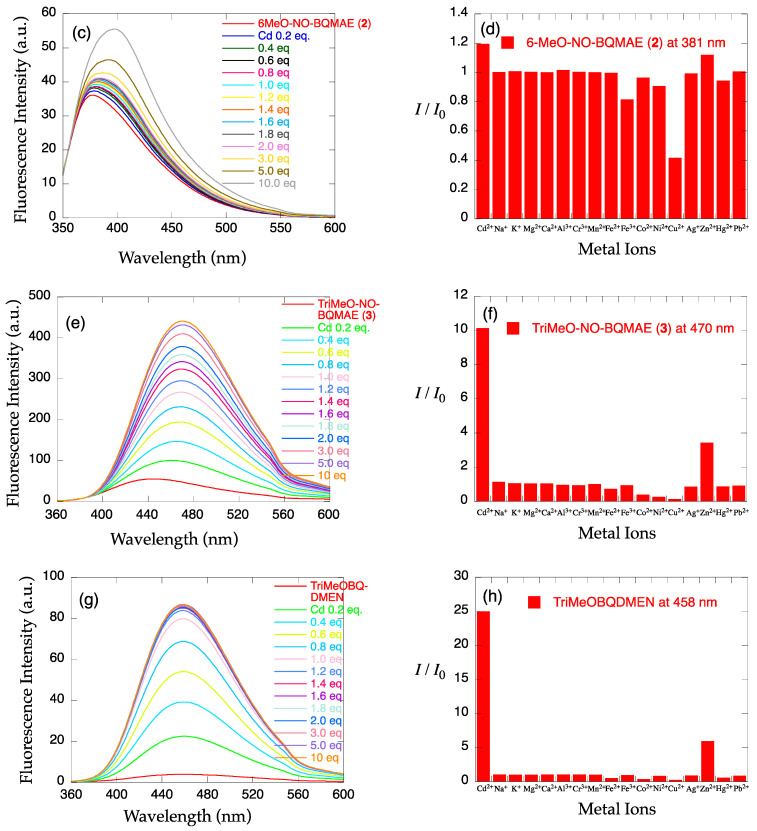
(**a**,**c**,**e**,**g**) Fluorescence spectral changes and (**b**,**d**,**f**,**h**) relative fluorescence intensity of 34 µM (**a**,**b**) *N,O*-BQMAE (**1**) (λ_ex_ = 317 nm), (**c**,**d**) 6-MeO*-N,O*-BQMAE (**2**) (λ_ex_ = 336 nm), (**e**,**f**) TriMeO-*N,O*-BQMAE (**3**) (λ_ex_ = 338 nm), and (**g**,**h**) TriMeOBQDMEN (λ_ex_ = 335 nm) in DMF-HEPES buffer (1:1, 50 mM HEPES, 0.1 M KCl, pH = 7.5) at 25 °C (**a**,**c**,**e**,**g**) in the presence of increasing concentrations of Cd^2+^ and (**b**,**d**,**f**,**h**) in the presence of 3 equiv. of metal ions. *I*_0_ is the emission intensity of free ligand.

The metal ion-induced fluorescence spectral changes of *N,O*-BQMAE derivatives **1**–**3** were examined in DMF-HEPES buffer (1:1, 50 mM HEPES, 0.1 M KCl, pH = 7.5) (Figure 1a–f). Absorbance spectral changes and excitation spectrum for **3** are shown in Figure S1. The previously reported N4 tetradentate fluorescent Cd^2+^ sensor TriMeOBQDMEN [29] was also re-examined in DMF-HEPES buffer (1:1, 50 mM HEPES, 0.1 M KCl, pH = 7.5) in this study for strict comparison (Figure 1g,h) because all measurements in the previous report for TriMeOBQDMEN were performed in DMF-H_2_O solution. As shown in Figure 1, only trimethoxy-substituted derivative **3** from the *N,O*-BQMAE derivatives exhibits distinct fluorescence enhancement upon addition of metal ions. The *N,O*-BQMAE (**1**) bearing unsubstituted quinolines does not respond to any metal ions except for a small response toward Cd^2+^, Zn^2+^, and Pb^2+^ (Figure 1b). The mono-methoxy derivative 6-MeO-*N,O*-BQMAE (**2**) only exhibits a Cu^2+^-induced fluorescence quenching (Figure 1d). The trimethoxy derivative TriMeO-*N,O*-BQMAE (**3**) affords apparent Cd^2+^-selective fluorescence enhancement over Zn^2+^ (*I*_Zn_/*I*_Cd_ = 34%) (Figure 1f), which is a bit diminished in selectivity in comparison with TriMeOBQDMEN under the same experimental condition (*I*_Zn_/*I*_Cd_ = 24%) (Figure 1h and Table 1).

The metal binding affinity (*K*_d_) was calculated from the non-linear fitting of titration curves with Cd^2+^ and Zn^2+^, indicating that TriMeO-*N,O*-BQMAE (**3**) has two orders lower metal binding ability than TriMeOBQDMEN (Figures S3 and S4 and Table 1). This is because the coordination ability of the nitrogen atom is higher than that of oxygen, but two orders of difference are quite significant for such a small modification.

### 2.3. Fluorescence Spectral Changes of N,N-BQMAE Derivatives ***4**–**6***

**Figure 2:**
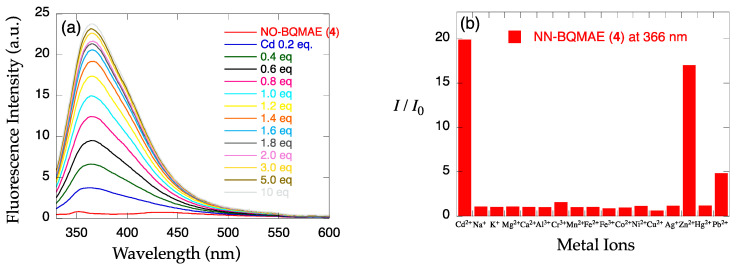

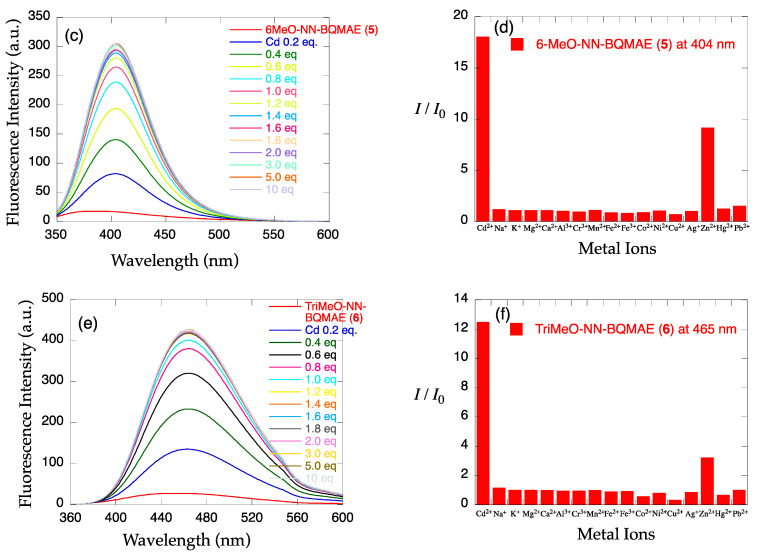
(**a**,**c**,**e**) Fluorescence spectral changes and (**b**,**d**,**f**) relative fluorescence intensity of 34 µM (**a**,**b**) *N,N*-BQMAE (**4**) (λ_ex_ = 317 nm), (**c**,**d**) 6-MeO*-N,N*-BQMAE (**5**) (λ_ex_ = 337 nm), and (**e**,**f**) TriMeO-*N,N*-BQMAE (**6**) (λ_ex_ = 340 nm) in DMF-HEPES buffer (1:1, 50 mM HEPES, 0.1 M KCl, pH = 7.5) at 25 °C (**a**,**c**,**e**) in the presence of increasing concentrations of Cd^2+^ and (**b**,**d**,**f**) in the presence of 3 equiv. of metal ions. *I*_0_ is the emission intensity of free ligand.

The metal ion-induced fluorescence spectral changes of *N,N*-BQMAE derivatives **4**–**6** were next examined (Figure 2). Absorbance spectral changes and excitation spectrum for **6** are shown in Figure S2. Because of the synthetic inconvenience of *N,O*-BQMAE derivatives **1**–**3** and disappointing fluorescence response of **1** and **2** toward metal ions due to the weak metal binding ability of these compounds, the other N3O1 ligands with a bis(2-quinolylmethyl)amine moiety were explored. As shown in Figure 2, even in the unsubstituted quinoline derivative *N,N*-BQMAE (**4**), metal-induced fluorescence enhancement was clearly observed, which is largely different from the *N,O*-isomer **1** and **2**. In the presence of a large excess of metal ions to assure the complete complexation, Zn^2+^ induces a slightly higher fluorescence intensity of **4** than Cd^2+^ (Figure S5). Other methoxy-substituted derivatives, including TriMeO-*N,O*-BQMAE (**3**) and TriMeOBQDMEN in the previous section, show complete metal binding in the presence of 3 equiv. of metal ions, and exhibit consistent preference of Cd^2+^ over Zn^2+^ (Figures S3, S4, S6 and S7). As the number of methoxy substituents increases, the fluorescence Cd^2+^ selectivity is improved (Figure 2b,d,f, and Table 1), and the metal binding affinity is enhanced (Figures S5–S7 and Table 1). The three trimethoxy-substituted derivatives, namely, TriMeO-*N,O*-BQMAE (**3**) (*K*_d(Zn)_ = ~10^−4^), TriMeO-*N,N*-BQMAE (**6**) (*K*_d(Zn)_ = ~10^−5^), and TriMeOBQDMEN (*K*_d(Zn)_ = ~10^−6^) exhibit approximately one order different metal binding affinities in this order (Table 1). Since the fluorescence metal ion selectivity of these compounds is essentially the same (*I*_Zn_/*I*_Cd_ = ~30%), this ligand library is a nice set of fluorescent sensors with different metal binding affinities, which is useful for convenient determination of Cd^2+^ concentration.

### 2.4. Effect of pH and Estimation of LOD

**Figure 3:**
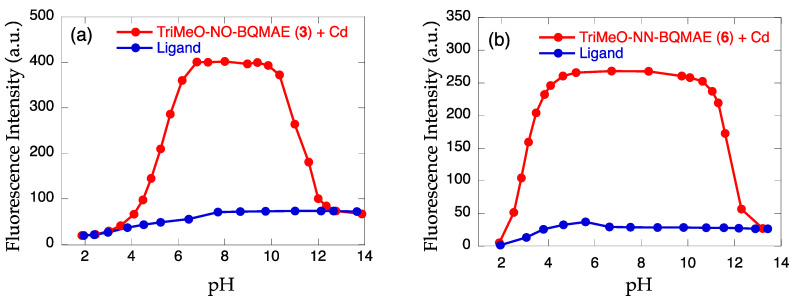
Effect of pH on the fluorescence intensity of (**a**) TriMeO-*N,O*-BQMAE (**3**) and (**b**) TriMeO-*N,N*-BQMAE (**6**) in the absence (blue) and presence (red) of 3 equiv. of Cd^2+^ in DMF-HEPES buffer (1:1, 50 mM HEPES, 0.1 M KCl, pH = 7.5) at 25 °C.

The effect of pH on the fluorescence intensity of the Cd^2+^ complex with TriMeO-*N,O*-BQMAE (**3**) and TriMeO-*N,N*-BQMAE (**6**) was examined (Figure 3). Protonation to the ligand at low pH region and coordination of hydroxide ion to Cd^2+^ at high pH region prevent the binding of Cd^2+^ to the probes. The pH window for fluorescent detection of Cd^2+^ is wider for **6** (Figure 3b) than **3** (Figure 3a), reflecting the difference in metal binding affinity of the ligands (Table 1). For **6**, fluorescence intensity is fairly stable between pH = 4 and pH = 11. The limit of detection (LOD) for **6** was estimated as low as 17 nM (Figure S8), which is comparable to the other tetradentate fluorescent Cd^2+^ probes in the literature [24,30,31]. This value (LOD = 17 nM) is lower than the environmental limit of water in Japan (3 ppb, 27 nM). Accordingly, the limit of quantitation (LOQ) is calculated to be 57 nM. The above properties of TriMeO-*N,N*-BQMAE (**6**) demonstrate the potential of this ligand for practical uses.

### 2.5. Fluorescence Lifetimes of N,O- and N,N-BQMAE Derivatives ***3**–**6*** and TriMeOBQDMEN

The fluorescence lifetimes (τ) for Cd^2+^ and Zn^2+^ complexes with *N,O*- and *N,N*-BQMAE derivatives **3**–**6** were measured in the presence of 2 equiv. of metal ions, in which most of the ligands form metal complexes. For comparison, TriMeOBQDMEN was also re-examined in DMF-HEPES buffer (1:1, 50 mM HEPES, 0.1 M KCl, pH = 7.5). Table 2 summarizes the results.

As discussed previously for TriMeOBQDMEN based on the investigation in DMF-H_2_O solution [29], the fluorescent Cd^2+^ selectivity of this ligand is explained by the difference in fluorescence lifetime in Cd^2+^ and Zn^2+^ complexes, in which a very long (τ = ~30 nsec) lifetime was observed exclusively for the Cd^2+^ complex. From the crystal structure of the Cd^2+^ complex with BQDMEN ([Cd_2_(µ-Cl)_2_(BQDMEN)_2_]^2+^), this long lifetime was assigned to be an intramolecular excimer emission from the highly stacked two quinoline rings with interligand interaction. Interestingly, in DMF-HEPES buffer (1:1, 50 mM HEPES, 0.1 M KCl, pH = 7.5), this long fluorescence lifetime was preserved for the TriMeOBQDMEN-Cd^2+^ complex (τ = 32 nsec) (Table 2). Here again, the Zn^2+^ complex with TriMeOBQDMEN exhibits a shorter lifetime (τ = 20 nsec) than Cd^2+^. These results strongly support that the fluorescence lifetime is a critical measure for the mechanism of fluorescence and structure of the metal complexes, which is independent of the solvent system used for measurements.

Similar distinct difference in fluorescence lifetimes of Cd^2+^ and Zn^2+^ complexes was found for TriMeO-*N*,*O*-BQMAE (**3**) (τ_Cd_ = 30 nsec and τ_Zn_ = 22 nsec). This is because the ligand structure of *N*,*O*-BQMAE resembles that of BQDMEN (Scheme 1) and rationally suggests the specific formation of a bis(µ-chloro) dinuclear cadmium complex for **3**. On the other hand, the *N,N*-BQMAE derivatives did not exhibit any differences in fluorescence lifetimes between Cd^2+^ and Zn^2+^ complexes. Even in the trimethoxy-substituted TriMeO-*N,N*-BQMAE (**6**), the lifetimes for Cd^2+^ and Zn^2+^ complexes are completely the same (τ_Cd_ = 18 nsec and τ_Zn_ = 17 nsec), and these values are too short to consider the excimer formation discussed above. These values (τ = ~20 nsec) are in good agreement with those for monomer complexes such as TriMeOTQTACN [28] (*N,N’,N’’*-tris(5,6,7-trimethoxy-2-quinolylmethyl)-1,4,7-triazacyclononane) (τ_Cd_ = 21 nsec and τ_Zn_ = 23 nsec) measured in MeOH-HEPES buffer (1:1, 50 mM HEPES, 0.1 M KCl, pH = 7.5). As a result, TriMeO-*N,O*-BQMAE (**3**) and TriMeO-*N,N*-BQMAE (**6**) adopt a different fluorescence mechanism possibly due to the different complex structure. Slightly diminished fluorescence quantum yield of **6** (ϕ_Cd_ = 0.23) in comparison to those of **3** (ϕ_Cd_ = 0.28) and BQDMEN (ϕ_Cd_ = 0.29) (Table 1) may reflect such differences. Although extensive trials of crystallization of Cd^2+^ complexes of **1**–**6** have been unsuccessful so far, the proton NMR indicates the coordination of quinoline and methoxy moieties to the metal center for *N,N*-BQMAE (**4**)-Cd^2+^ complex, affording the chemical shift changes (Figure S9). The characteristic peak splitting of the methylene signal around 4.4–4.7 ppm also supports the coordination of 2-aminomethylquinoline moiety to the metal center.

At the present stage, the details in the fluorescent Cd^2+^/Zn^2+^ discrimination mechanism of TriMeO-*N,N*-BQMAE (**6**) is still unknown, but the dynamic equilibrium of mononuclear ([Cd(L)Cl]^+^) and µ-chloro-bridged dinuclear ([Cd_2_(µ-Cl)_2_(L)_2_]^2+^) cadmium complexes needs to be considered. To answer these critical questions, related projects using other ligand systems with different skeletons are now in progress in our laboratory.

## 3. Experimental

### 3.1. Materials and Methods

All reagents and solvents used for the preparation of probe molecules were obtained from commercial sources and used as received. DMF (*N,N*-dimethylformamide) used for spectroscopic measurements was of spectral grade (Dojin, Spectrosol). All aqueous solutions were prepared using Milli-Q water (Millipore, Merck, Germany). ^1^H/^13^C NMR spectra were recorded on a JEOL (Akishima, Japan) AL-400 (400/100 MHz) spectrometer and referenced to internal Si(CH_3_)_4_ or solvent signal. Elemental analyses were recorded on J-Science (Kyoto, Japan) JM-10 micro corder. UV-vis and fluorescence spectra were measured on a Jasco (Hachioji, Japan) V-660 spectrophotometer and Jasco (Hachioji, Japan) FP-6300 spectrofluorometer, respectively. Fluorescence quantum yields were measured on a HAMAMATSU photonics (Hamamatsu, Japan) C9920-02 absolute PL quantum yield measurement system. Fluorescence lifetimes were measured on a HORIBA fluorescence lifetime system Tempro equipped with a 370, 400, 460, or 490 nm bandpath filter. TriMeOBQDMEN [29] was prepared as described previously. CAUTION: Perchlorate salts of metal complexes with organic ligands are potentially explosive. All due precautions should be taken.

### 3.2. Synthesis of N,O-Bis(2-quinolylmethyl)-2-methylaminoethanol (N,O-BQMAE (***1***))

The mixture of 2-methylaminoethanol (37.6 mg, 0.501 mmol), 2-chloromethylquinoline (89.8 mg, 0.506 mmol), potassium carbonate (71.4 mg, 0.517 mmol), and potassium iodide (84.5 mg, 0.509 mmol) in dry acetonitrile (15 mL) was refluxed for 23 h. After the reaction mixture was cooled to room temperature, solvent was removed under reduced pressure, and organic materials were extracted with dichloromethane–water. The organic layer was dried and evaporated to afford *N*-(2-quinolylmethyl)-2-methylaminoethanol as yellow oil (106 mg, 0.490 mmol, 98%).

This material (200 mg, 0.925 mmol) was dissolved in THF (4 mL) and cooled on ice, then *n*-butyllithium (1.6 M solution in hexane, 869 µL, 1.39 mmol) was added slowly through a syringe. After stirring for 30 min on ice, 2-chloromethylquinoline (205 mg, 1.15 mmol) in THF (2 mL) was added slowly through a syringe. The reaction mixture was warmed to room temperature and refluxed for 2 h. After the reaction, solvent was removed under reduced pressure, and organic materials were extracted with dichloromethane–water. The organic layer was dried and evaporated, and the residue was purified by alumina column chromatography using chloroform containing 0.1% methanol (*R*_f_ = 0.25) as an eluent. The product was further purified by recycling GPC to afford *N,O*-bis(2-quinolylmethyl)-2-methylaminoethanol (*N,O*-BQMAE (**1**)) as yellow oil (21.3 mg, 0.0596 mmol, 6%).

^1^H NMR (CDCl_3_, 400 MHz): δ (ppm) 8.03–8.15 (m, 4H, quinoline-H4 and H8), 7.78–7.82 (m, 2H, quinoline-H5), 7.67–7.72 (m, 3H, quinoline-H3 and H6), 7.62 (d, *J* = 8.3 Hz, 1H, quinoline-H3), 7.49–7.54 (m, 2H, quinoline-H7), 4.84 (s, 2H, OC*H_2_*Ar), 3.94 (s, 2H, NC*H_2_*Ar), 3.77 (t, *J* = 5.6 Hz, 2H, OC*H_2_*CH_2_), 2.85 (t, *J* = 5.6 Hz, 2H, NC*H_2_*CH_2_), 2.41 (s, 3H, NC*H_3_*).

^13^C NMR (CDCl_3_, 100 MHz): δ (ppm) 160.2, 259.3, 147.6, 147.5, 136.7, 136.3, 129.5, 129.3, 129.0, 128.9, 127.6, 127.5, 126.2, 126.1, 121.1, 119.3, 74.6, 69.1, 64.8, 57.0, 43.3.

HRMS (ESI) *m/z*: [**1** + Na]^+^ calcd. for C_23_H_23_N_3_ONa 380.17388. Found 380.17026.

### 3.3. Synthesis of N,O-Bis(6-methoxy-2-quinolylmethyl)-2-methylaminoethanol (6-MeO-N,O-BQMAE (***2***))

The mixture of 2-methylaminoethanol (62.8 mg, 0.836 mmol), 6-methoxy-2-chloromethylquinoline (154 mg, 0.742 mmol), potassium carbonate (103 mg, 0.745 mmol), and potassium iodide (142 mg, 0.855 mmol) in dry acetonitrile (20 mL) was refluxed for 23 h. After the reaction mixture was cooled to room temperature, solvent was removed under reduced pressure, and organic materials were extracted with dichloromethane–water. The organic layer was dried and evaporated to afford *N*-(6-methoxy-2-quinolylmethyl)-2-methylaminoethanol as yellow oil in quantitative yield.

This material (183 mg, 0.742 mmol) was dissolved in THF (4 mL) and cooled on ice, then *n*-butyllithium (1.6 M solution in hexane, 798 µL, 1.28 mmol) was added slowly through a syringe. After stirring for 30 min on ice, 6-methoxy-2-chloromethylquinoline (177 mg, 0.852 mmol) in THF (2 mL) was added slowly through a syringe. The reaction mixture was warmed to room temperature and refluxed for 2 h. After the reaction, solvent was removed under reduced pressure, and organic materials were extracted with dichloromethane–water. The organic layer was dried and evaporated, and the residue was purified by alumina column chromatography using chloroform containing 0.3% methanol (*R*_f_ = 0.3) as an eluent. The product was further purified by recycling GPC to afford *N,O*-bis(6-methoxy-2-quinolylmethyl)-2-methylaminoethanol (6-MeO-*N,O*-BQMAE (**2**)) as brown oil (54.8 mg, 0.131 mmol, 18%).

^1^H NMR (CDCl_3_, 400 MHz): δ (ppm) 7.95–8.00 (m, 3H, quinoline-H4 and H8), 7.89 (d, *J* = 8.3 Hz, 1H, quinoline-H8), 7.29–7.37 (m, 4H, quinoline-H3 and H7), 7.04 (d, *J* = 2.9 Hz, 1H, quinoline-H5), 7.01 (d, *J* = 2.9 Hz, 1H, quinoline-H5), 4.68 (t, *J* = 7.3 Hz, 1H, OC*H_2_*Ar), 4.0–4.5 (br., 1H, OC*H_2_*Ar), 3.92 (s, 3H, OC*H_3_*), 3.90 (s, 3H, OC*H_3_*), 3.56–3.76 (m, 4H, NC*H_2_*Ar and OC*H_2_*CH_2_), 2.91–2.98 (m, 1H, NC*H_2_*CH_2_), 2.61–2.65 (m, 1H, NC*H_2_*CH_2_), 2.36 (s, 3H, NC*H_3_*).

^13^C NMR (CDCl_3_, 100 MHz): δ (ppm) 158.1, 157.6, 157.3, 157.0, 143.8, 143.5, 134.9, 134.6, 130.8, 130.1, 128.1, 127.6, 122.8, 122.0, 121.9, 105.1, 105.1, 68.6, 58.7, 55.53, 55.48, 55.42, 38.5, 38.1.

HRMS (ESI) *m/z*: [**2** + H]^+^ calcd. for C_25_H_28_N_3_O_3_ 418.21307. Found 418.22029.

### 3.4. Synthesis of N,O-Bis(5,6,7-trimethoxy-2-quinolylmethyl)-2-methylaminoethanol (TriMeO-N,O-BQMAE (***3***))

The mixture of 2-methylaminoethanol (59.0 mg, 0.786 mmol), 5,6,7-trimethoxy-2-chloromethylquinoline (199 mg, 0.743 mmol), potassium carbonate (105 mg, 0.760 mmol), and potassium iodide (125 mg, 0.753 mmol) in dry acetonitrile (20 mL) was refluxed for 23 h. After the reaction mixture was cooled to room temperature, solvent was removed under reduced pressure, and organic materials were extracted with dichloromethane–water. The organic layer was dried and evaporated to afford *N*-(5,6,7-trimethoxy-2-quinolylmethyl)-2-methylaminoethanol as yellow oil in quantitative yield.

This material (220 mg, 0.718 mmol) was dissolved in THF (4 mL) and cooled on ice, then *n*-butyllithium (1.6 M solution in hexane, 675 µL, 1.08 mmol) was added slowly through a syringe. After stirring for 30 min on ice, 5,6,7-trimethoxy-2-chloromethylquinoline (195 mg, 0.728 mmol) in THF (2 mL) was added slowly through a syringe. The reaction mixture was warmed to room temperature and refluxed for 2 h. After the reaction, solvent was removed under reduced pressure, and organic materials were extracted with dichloromethane–water. The organic layer was dried and evaporated, and the residue was purified by alumina column chromatography using chloroform containing 0.3% methanol (*R*_f_ = 0.4) as an eluent. The product was further purified by recycling GPC to afford *N,O*-bis(5,6,7-trimethoxy-2-quinolylmethyl)-2-methylaminoethanol (TriMeO-*N,O*-BQMAE (**3**)) as brown oil (73.2 mg, 0.136 mmol, 19%).

^1^H NMR (CDCl_3_, 400 MHz): δ (ppm) 8.34 (d, *J* = 8.8 Hz, 1H, quinoline-H4), 8.31 (d, *J* = 8.3 Hz, 1H, quinoline-H4), 7.53 (d, *J* = 8.8 Hz, 1H, quinoline-H3), 7.47 (d, *J* = 8.3 Hz, 1H, quinoline-H3), 7.24 (s, 1H, quinoline-H8), 7.21 (s, 1H, quinoline-H8), 4.78 (s, 2H, OC*H_2_*Ar), 4.06 (s, 6H, OC*H_3_*), 4.00 (s, 6H, OC*H_3_*), 3.98 (s, 6H, OC*H_3_*), 3.89 (s, 2H, NC*H_2_*Ar), 3.77 (t, *J* = 5.6 Hz, 2H, OC*H_2_*CH_2_), 2.83 (t, *J* = 5.6 Hz, 2H, NC*H_2_*CH_2_), 2.40 (s, 3H, NC*H_3_*).

^13^C NMR (CDCl_3_, 100 MHz): δ (ppm) 158.4, 155.9, 155.7, 146.9, 145.3, 140.4, 130.9, 130.6, 118.8, 118.4, 118.1, 117.0, 103.9, 130.7, 74.5, 68.9, 64.7, 61.5, 61.2, 56.9, 56.0, 43.2.

Anal. Calcd for C_29_H_36.4_N_3_O_7.7_ (**3**·0.7H_2_O): H, 6.67; C, 63.31; N, 7.64. Found: H, 6.36; C, 63.16; N, 7.36.

HRMS (ESI) *m/z*: [**3** + H]^+^ calcd. for C_29_H_36_N_3_O_7_ 538.25532. Found 538.25830.

### 3.5. Synthesis of N,N-Bis(2-quinolylmethyl)-2-methoxyethylamine (N,N-BQMAE (***4***))

The mixture of 2-methoxyethylamine (22.4 mg, 0.298 mmol), 2-chloromethylquinoline (88.6 mg, 0.499 mmol), and potassium carbonate (69.1 mg, 0.500 mmol) in dry acetonitrile (10 mL) was refluxed for 21 h. After the reaction mixture was cooled to room temperature, solvent was removed under reduced pressure, and organic materials were extracted with dichloromethane–water. The organic layer was dried and evaporated to afford *N,N*-bis(2-quinolylmethyl)-2-methoxyethylamine (*N,N*-BQMAE (**4**)) as a yellow-white solid in quantitative yield.

^1^H NMR (CDCl_3_, 400 MHz): δ (ppm) 8.13 (d, *J* = 8.7 Hz, 2H, quinoline-H4), 8.04 (d, *J* = 8.3 Hz, 2H, quinoline-H8), 7.76–7.81 (m, 4H, quinoline-H3 and H5), 7.68 (dt, *J* = 1.0, 8.3 Hz, 2H, quinoline-H6), 7.50 (t, *J* = 7.4 Hz, 2H, quinoline-H7), 4.11 (s, 4H, NC*H_2_*Ar), 3.56 (t, *J* = 5.9 Hz, 2H, OC*H_2_*CH_2_), 3.27 (s, 3H, OC*H_3_*), 2.89 (t, *J* = 5.9 Hz, 2H, NC*H_2_*CH_2_).

^13^C NMR (CDCl_3_, 100 MHz): δ (ppm) 160.5, 147.5, 136.2, 129.3, 128.9, 127.4, 127.3, 126.0, 121.0, 71.0, 61.8, 58.6, 53.7.

Anal. Calcd for C_23_H_23.4_N_3_O_1.2_ (**4**·0.2H_2_O): H, 6.53; C, 76.51; N, 11.64. Found: H, 6.55; C, 76.56; N, 11.72.

HRMS (ESI) *m/z*: [**4** + Na]^+^ calcd. for C_23_H_23_N_3_ONa 380.17388. Found 380.18173.

### 3.6. Synthesis of N,N-Bis(6-methoxy-2-quinolylmethyl)-2-methoxyethylamine (6-MeO-N,N-BQMAE (***5***))

The mixture of 2-methoxyethylamine (38.3 mg, 0.510 mmol), 6-methoxy-2-chloromethylquinoline (215 mg, 1.04 mmol), and potassium carbonate (138 mg, 0.998 mmol) in dry acetonitrile (20 mL) was refluxed for 21 h. After the reaction mixture was cooled to room temperature, solvent was removed under reduced pressure, and organic materials were extracted with dichloromethane–water. The organic layer was dried and evaporated to afford *N,N*-bis(6-methoxy-2-quinolylmethyl)-2-methoxyethylamine (6-MeO-*N,N*-BQMAE (**5**)) as yellow oil (208 mg, 0.498 mmol, 96%).

^1^H NMR (CDCl_3_, 400 MHz): δ (ppm) 8.01 (d, *J* = 8.8 Hz, 2H, quinoline-H4), 7.94 (d, *J* = 9.3 Hz, 2H, quinoline-H8), 7.70 (d, *J* = 8.3 Hz, 2H, quinoline-H3), 7.33 (dd, *J* = 2.9, 9.2 Hz, 2H, quinoline-H7), 7.05 (d, *J* = 2.9 Hz, 2H, quinoline-H5), 4.05 (s, 4H, NC*H_2_*Ar), 3.92 (s, 6H, OC*H_3_*), 3.55 (t, *J* = 5.6 Hz, 2H, OC*H_2_*CH_2_), 3.26 (s, 3H, OC*H_3_*), 2.87 (t, *J* = 5.6 Hz, 2H, NC*H_2_*CH_2_).

^13^C NMR (CDCl_3_, 100 MHz): δ (ppm) 157.8, 157.4, 134.4, 135.1, 130.3, 128.2, 121.8, 121.4, 105.1, 70.9, 61.5, 58.6, 55.4, 53.6.

Anal. Calcd for C_25_H_28_N_3_O_3.5_ (**5**·0.5H_2_O): H, 6.62; C, 70.40; N, 9.85. Found: H, 6.42; C, 70.15; N, 9.80.

HRMS (ESI) *m/z*: [**5** + Na]^+^ calcd. for C_25_H_27_N_3_O_3_Na 440.19501. Found 440.20330.

### 3.7. Synthesis of N,N-Bis(5,6,7-trimethoxy-2-quinolylmethyl)-2-methoxyethylamine (TriMeO-N,N-BQMAE (***6***))

The mixture of 2-methoxyethylamine (27.3 mg, 0.363 mmol), 5,6,7-trimethoxy-2-chloromethylquinoline (201 mg, 0.751 mmol), and potassium carbonate (103 mg, 0.745 mmol) in dry acetonitrile (15 mL) was refluxed for 21 h. After the reaction mixture was cooled to room temperature, solvent was removed under reduced pressure, and organic materials were extracted with dichloromethane–water. The organic layer was dried and evaporated to afford *N,N*-bis(5,6,7-trimethoxy-2-quinolylmethyl)-2-methoxyethylamine (TriMeO-*N,N*-BQMAE (**6**)) as yellow oil in quantitative yield.

^1^H NMR (CDCl_3_, 400 MHz): δ (ppm) 8.31 (d, *J* = 8.3 Hz, 2H, quinoline-H4), 7.62 (d, *J* = 8.8 Hz, 2H, quinoline-H3), 7.20 (s, 2H, quinoline-H8), 4.04 (s, 10H, NC*H_2_*Ar and OC*H_3_*), 3.98 (s, 6H, OC*H_3_*), 3.97 (s, 6H, OC*H_3_*), 3.57 (t, *J* = 5.6 Hz, 2H, OC*H_2_*CH_2_), 3.29 (s, 3H, OC*H_3_*), 2.87 (t, *J* = 5.6 Hz, 2H, NC*H_2_*CH_2_).

^13^C NMR (CDCl_3_, 100 MHz): δ (ppm) 159.9, 155.7, 146.9, 145.2, 140.4, 130.5, 118.7, 118.1, 103.9, 71.0, 61.7, 61.5, 61.1, 58.7, 56.0, 53.7.

Anal. Calcd for C_29.3_H_35.3_Cl_0.9_N_3_O_7_ (**6**·0.3CHCl_3_): H, 6.20; C, 61.37; N, 7.33. Found: H, 6.05; C, 61.66; N, 7.34.

HRMS (ESI) *m/z*: [**6** + Na]^+^ calcd. for C_29_H_35_N_3_O_7_Na 560.23727. Found 560.24627.

## 4. Conclusions

The newly designed six bisquinoline-based N3O1 tetradentate ligands with 2-aminoethanol backbone were examined as a fluorescent Cd^2+^ sensor. This ligand library includes *N,O*-BQMAE derivatives **1**–**3** and *N,O*-BQMAE derivatives **4**–**6**, which differ in the position of two quinoline moieties and the number of methoxy substituents on the quinoline rings. Judging from the moderately good fluorescent Cd^2+^ selectivity over Zn^2+^ (*I*_Zn_/*I*_Cd_ = ~30%) and wide range of metal binding affinity (two orders differences in *K*_d_), the trimethoxy derivatives TriMeO-*N,O*-BQMAE (**3**) and TriMeO-*N,N*-BQMAE (**6**), as well as previously reported TriMeOBQDMEN, are regarded as a potential set of fluorescent ligands with different metal binding affinities useful for convenient quantification of Cd^2+^ in solution.

## Figures and Tables

**Table 1:** Fluorescent and Thermodynamic Properties for Cd^2+^ and Zn^2+^ Complexes of BQMAE Derivatives **3**–**6** and TriMeOBQDMEN ^a^.

Ligand	λ_ex_ (nm)	λ_em_ (nm) ^b^	*I*_Zn_/*I*_Cd_^c^	ϕ_Cd_	*K*_d_ for Cd^2+^ Complex (M)	*K*_d_ for Zn^2+^ Complex (M)
TriMeO-*N,O*-BQMAE (**3**)	338	470	34%	0.281 ^d^	(1.4 ± 0.2) × 10^−5^	(8.2 ± 0.4) × 10^−5^
*N,N*-BQMAE (**4**)	317	366	87%	0.018 ^d^	(8.1 ± 1.5) × 10^−6^	(5.2 ± 0.3) × 10^−5^
6-MeO-*N,N*-BQMAE (**5**)	337	404	51%	0.171 ^d^	(6.4 ± 0.3) × 10^−7^	(1.6 ± 0.2) × 10^−5^
TriMeO-*N,N*-BQMAE (**6**)	340	465	26%	0.228 ^d^	~10^−7^	(1.5 ± 0.2) × 10^−5^
TriMeOBQDMEN	335	458	24% (41% ^e^)	0.293 ^c,e^	(2.6 ± 1.0) × 10^−7^	(2.7 ± 0.5) × 10^−6^

^a^ In DMF-HEPES buffer (1:1, 50 mM HEPES, 0.1 M KCl, pH = 7.5) at 25 °C. ^b^ For Cd^2+^ complex. ^c^ In the presence of 3 equiv. of metal ions. ^d^ In the presence of 10 equiv. of metal ions. ^e^ In DMF-H_2_O (1:1) [29].

**Table 2:** Fluorescence Lifetimes for Cd^2+^ and Zn^2+^ Complexes of BQMAE Derivatives **3**–**6** and TriMeOBQDMEN ^a,b^.

Ligand	Metal Ion	λ_em_ (nm)	BPF (nm) ^c^	τ (nsec) ^d^
TriMeO-*N,O*-BQMAE (**3**)	—	—	460	1.2 (17%), 4.3 (74%), 14.5 (9%)
	Cd^2+^	470	460	2.6 (5%), 11.5 (14%), 29.7 (81%)
	Zn^2+^	484	460	2.1 (14%), 7.5 (34%), 22 (52%)
*N,N*-BQMAE (**4**)	—	—	370	1.2 (14%), 5.5 (40%)
	Cd^2+^	364	370	5.4 (20%)
	Zn^2+^	370	370	1.0 (43%), 5.3 (22%)
6-MeO-*N,N*-BQMAE (**5**)	—	—	400	2.7 (45%), 6.0 (46%)
	Cd^2+^	404	400	1.1 (5%), 3.0 (1%), 6.2 (94%)
	Zn^2+^	408	400	3.4 (27%), 6.8 (66%)
TriMeO-*N,N*-BQMAE (**6**)	—	—	460	1.2 (34%), 4.0 (24%), 16.8 (42%)
	Cd^2+^	465	460	1.6 (2%), 8.5 (7%), 17.7 (91%)
	Zn^2+^	480	490	1.2 (12%), 7.4 (33%), 16.5 (54%)
TriMeOBQDMEN	—	—	460	1.1 (29%), 4.3 (44%), 21.4 (27%)
	Cd^2+^	472	460	3.5 (1%), 14.6 (5%), 31.8 (91%)
	Zn^2+^	487	490	1.7 (2%), 7.8 (9%), 20.2 (88%)

^a^ In DMF-HEPES buffer (1:1, 50 mM HEPES, 0.1 M KCl, pH = 7.5). ^b^ In the presence of 2 equiv. of metal ions. ^c^ Bandpath filter used (±10 nm). ^d^ Components with extremely short lifetimes (<1 nsec) were omitted.

## Data Availability

Data are contained within the article and supplementary materials.

## Supplementary Materials

The following supporting information can be downloaded at: https://www.mdpi.com/article/10.3390/molecules29020369/s1, Scheme S1: Synthesis of ligands **1**–**6**; Figure S1: Absorbance spectral changes of 34 µM *N,O*-BQMAE (**1**), 6-MeO*-N,O*-BQMAE (**2**), and TriMeO-*N,O*-BQMAE (**3**) in DMF-HEPES buffer (1:1, 50 mM HEPES, 0.1 M KCl, pH = 7.5) at 25 °C in the presence of increasing concentrations of Cd^2+^; Figure S2: Absorbance spectral changes of 34 µM *N,N*-BQMAE (**4**), 6-MeO*-N,N*-BQMAE (**5**), and TriMeO-*N,N*-BQMAE (**6**) in DMF-HEPES buffer (1:1, 50 mM HEPES, 0.1 M KCl, pH = 7.5) at 25 °C in the presence of increasing concentrations of Cd^2+^; Figure S3: Estimation of dissociation constants (*K*_d_) for TriMeO-*N,O*-BQMAE (**3**) with Cd^2+^ and Zn^2+^ in DMF-HEPES buffer (1:1, 50 mM HEPES, 0.1 M KCl, pH = 7.5) at 25 °C; Figure S4: Estimation of dissociation constants (*K*_d_) for TriMeOBQDMEN with Cd^2+^ and Zn^2+^ in DMF-HEPES buffer (1:1, 50 mM HEPES, 0.1 M KCl, pH = 7.5) at 25 °C; Figure S5: Estimation of dissociation constants (*K*_d_) for *N,N*-BQMAE (**4**) with Cd^2+^ and Zn^2+^ in DMF-HEPES buffer (1:1, 50 mM HEPES, 0.1 M KCl, pH = 7.5) at 25 °C; Figure S6: Estimation of dissociation constants (*K*_d_) for 6-MeO-*N,N*-BQMAE (**5**) with Cd^2+^ and Zn^2+^ in DMF-HEPES buffer (1:1, 50 mM HEPES, 0.1 M KCl, pH = 7.5) at 25 °C; Figure S7: Estimation of dissociation constants (*K*_d_) for TriMeO-*N,N*-BQMAE (**6**) with Cd^2+^ and Zn^2+^ in DMF-HEPES buffer (1:1, 50 mM HEPES, 0.1 M KCl, pH = 7.5) at 25 °C; Figure S8: Estimation of LOD (limit of detection) for Cd^2+^ with TriMeO-*N,N*-BQMAE (**6**) in DMF-HEPES buffer (1:1, 50 mM HEPES, 0.1 M KCl, pH = 7.5) at 25 °C; Figure S9: ^1^H NMR spectrum of Cd^2+^ complex of *N,N*-BQMAE (**4**) in DMSO-*d*_6_; Figure S10: ^1^H/^13^C NMR spectrum of *N,O*-BQMAE (**1**) in CDCl_3_; Figure S11: ^1^H/^13^C NMR spectrum of 6-MeO-*N,O*-BQMAE (**2**) in CDCl_3_; Figure S12: ^1^H/^13^C NMR spectrum of TriMeO-*N,O*-BQMAE (**3**) in CDCl_3_; Figure S13: ^1^H/^13^C NMR spectrum of *N,N*-BQMAE (**4**) in CDCl_3_; Figure S14: ^1^H/^13^C NMR spectrum of 6-MeO-*N,N*-BQMAE (**5**) in CDCl_3_; Figure S15: ^1^H/^13^C NMR spectrum of TriMeO-*N,N*-BQMAE (**6**) in CDCl_3_.
