# Supplementary material for: Cd2+-Selective Fluorescence Enhancement of Bisquinoline Derivatives with 2-Aminoethanol Skeleton
Source: Molecules. 2024 Jan 11;29(2):369. doi: 10.3390/molecules29020369 (PMC10820404; doi:10.3390/molecules29020369)
Supplement: Supplementary file 1 [file molecules-29-00369-s001.zip › molecules-2774303-supplementary.pdf]

Supplementary Materials for

Cd^{2+}-Selective Fluorescence Enhancement of Bisquinoline Derivatives with 2-Aminoethanol Skeleton

Yuji Mikata [1,2,3,4,*], Aya Tsuruta [3], Hinata Koike [4], Sunao Shoji [1,4], and Hideo Konno [5]

[1]*Laboratory for Molecular & Functional Design, Department of Engineering, Nara Women's University, Nara 630-8506, Japan; mikata@nara-wu.ac.jp*

[2]*KYOUSEI Science Center, Nara Women's University, Nara 630-8506, Japan*

[3]*Department of Chemistry, Biology, and Environmental Science, Faculty of Science, Nara Women's University, Nara 630-8506, Japan*

[4]*Cooperative Major in Human Centered Engineering, Nara Women's University, Nara 630-8506, Japan*

[5]*National Institute of Advanced Industrial Science and Technology (AIST), 1-1-1 Higashi, Tsukuba, Ibaraki 305-8565, Japan*

**Correspondence: mikata@nara-wu.ac.jp*

INDEX

Preparation of ligands··········S4

Scheme S1. Synthesis of ligands **1-6**.

Absorbance and excitation spectra··········S5-S6

Figure S1. Absorbance spectral changes of 34 μM *N,O*-BQMAE (**1**), 6-MeO-*N,O*-BQMAE (**2**), and TriMeO-*N,O*-BQMAE (**3**) in DMF-HEPES buffer (1:1, 50 mM HEPES, 0.1 M KCl, pH = 7.5) at 25 °C in the presence of increasing concentrations of Cd^{2+}. Excitation spectrum of 34 μM TriMeO-*N,O*-BQMAE (**3**) in DMF-HEPES buffer (1:1, 50 mM HEPES, 0.1 M KCl, pH = 7.5) at 25 °C (λ_{em} = 445 nm).

Figure S2. Absorbance spectral changes of 34 μM *N,N*-BQMAE (**4**), 6-MeO-*N,N*-BQMAE (**5**), and TriMeO-*N,N*-BQMAE (**6**) in DMF-HEPES buffer (1:1, 50 mM HEPES, 0.1 M KCl, pH = 7.5) at 25 °C in the presence of increasing concentrations of Cd^{2+}. Excitation spectrum of 34 μM TriMeO-*N,N*-BQMAE (**6**) in DMF-HEPES buffer (1:1, 50 mM HEPES, 0.1 M KCl, pH = 7.5) at 25 °C (λ_{em} = 450 nm).

Estimation of dissociation constants and LOD··········S7-S12

Figure S3. Estimation of dissociation constants (K_d) for TriMeO-*N,O*-BQMAE (**3**) with Cd^{2+} and Zn^{2+} in DMF-HEPES buffer (1:1, 50 mM HEPES, 0.1 M KCl, pH = 7.5) at 25 °C.

Figure S4. Estimation of dissociation constants (K_d) for TriMeOBQDMEN with Cd^{2+} and Zn^{2+} in DMF-HEPES buffer (1:1, 50 mM HEPES, 0.1 M KCl, pH = 7.5) at 25 °C.

Figure S5. Estimation of dissociation constants (K_d) for *N,N*-BQMAE (**4**) with Cd^{2+} and Zn^{2+} in DMF-HEPES buffer (1:1, 50 mM HEPES, 0.1 M KCl, pH = 7.5) at 25 °C.

Figure S6. Estimation of dissociation constants (K_d) for 6-MeO-*N,N*-BQMAE (**5**) with Cd^{2+} and Zn^{2+} in DMF-HEPES buffer (1:1, 50 mM HEPES, 0.1 M KCl, pH = 7.5) at 25 °C.

Figure S7. Estimation of dissociation constants (K_d) for TriMeO-*N,N*-BQMAE (**6**) with Cd^{2+} and Zn^{2+} in DMF-HEPES buffer (1:1, 50 mM HEPES, 0.1 M KCl, pH = 7.5) at 25 °C.

Figure S8. Estimation of LOD (limit of detection) for Cd^{2+} with TriMeO-*N,N*-BQMAE (**6**) in DMF-HEPES buffer (1:1, 50 mM HEPES, 0.1 M KCl, pH = 7.5) at 25 °C.

***$^{1}H/^{13}C$ NMR spectra*···S13-S19**

Figure S9. ^{1}H NMR spectra of Cd^{2+} complex and free ligand of *N,N*-BQMAE (**4**) in DMSO-d_6.

Figure S10. $^{1}H/^{13}C$ NMR spectrum of *N,O*-BQMAE (**1**) in $CDCl_3$.

Figure S11. $^{1}H/^{13}C$ NMR spectrum of 6-MeO-*N,O*-BQMAE (**2**) in $CDCl_3$.

Figure S12. $^{1}H/^{13}C$ NMR spectrum of TriMeO-*N,O*-BQMAE (**3**) in $CDCl_3$.

Figure S13. $^{1}H/^{13}C$ NMR spectrum of *N,N*-BQMAE (**4**) in $CDCl_3$.

Figure S14. $^{1}H/^{13}C$ NMR spectrum of 6-MeO-*N,N*-BQMAE (**5**) in $CDCl_3$.

Figure S15. $^{1}H/^{13}C$ NMR spectrum of TriMeO-*N,N*-BQMAE (**6**) in $CDCl_3$.

Scheme S1

(a)

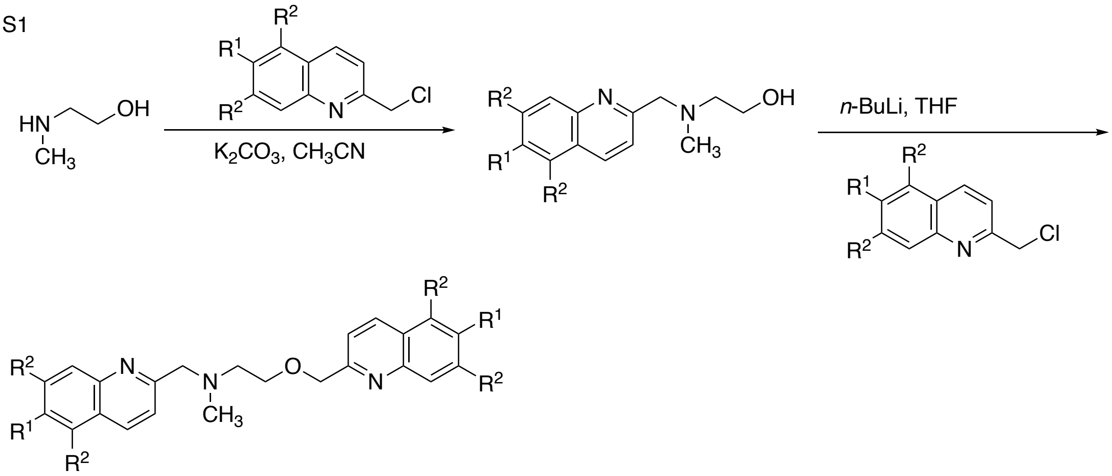

$R^1 = R^2 = H$	: *N,O*-BQMAE (**1**)	6% for 2 steps
$R^1 = OCH_3$, $R^2 = H$	: 6-MeO-*N,O*-BQMAE (**2**)	18% for 2 steps
$R^1 = R^2 = OCH_3$	: TriMeO-*N,O*-BQMAE (**3**)	19% for 2 steps

(b)

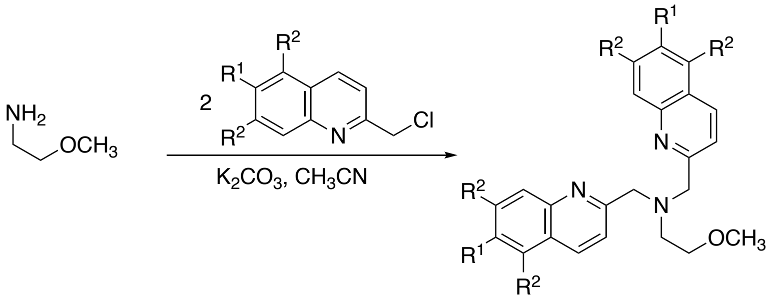

$R^1 = R^2 = H$	: *N,N*-BQMAE (**4**)	quant.
$R^1 = OCH_3$, $R^2 = H$	: 6-MeO-*N,N*-BQMAE (**5**)	96%
$R^1 = R^2 = OCH_3$	: TriMeO-*N,N*-BQMAE (**6**)	quant.

Scheme S1. Synthesis of ligands **1-6**.

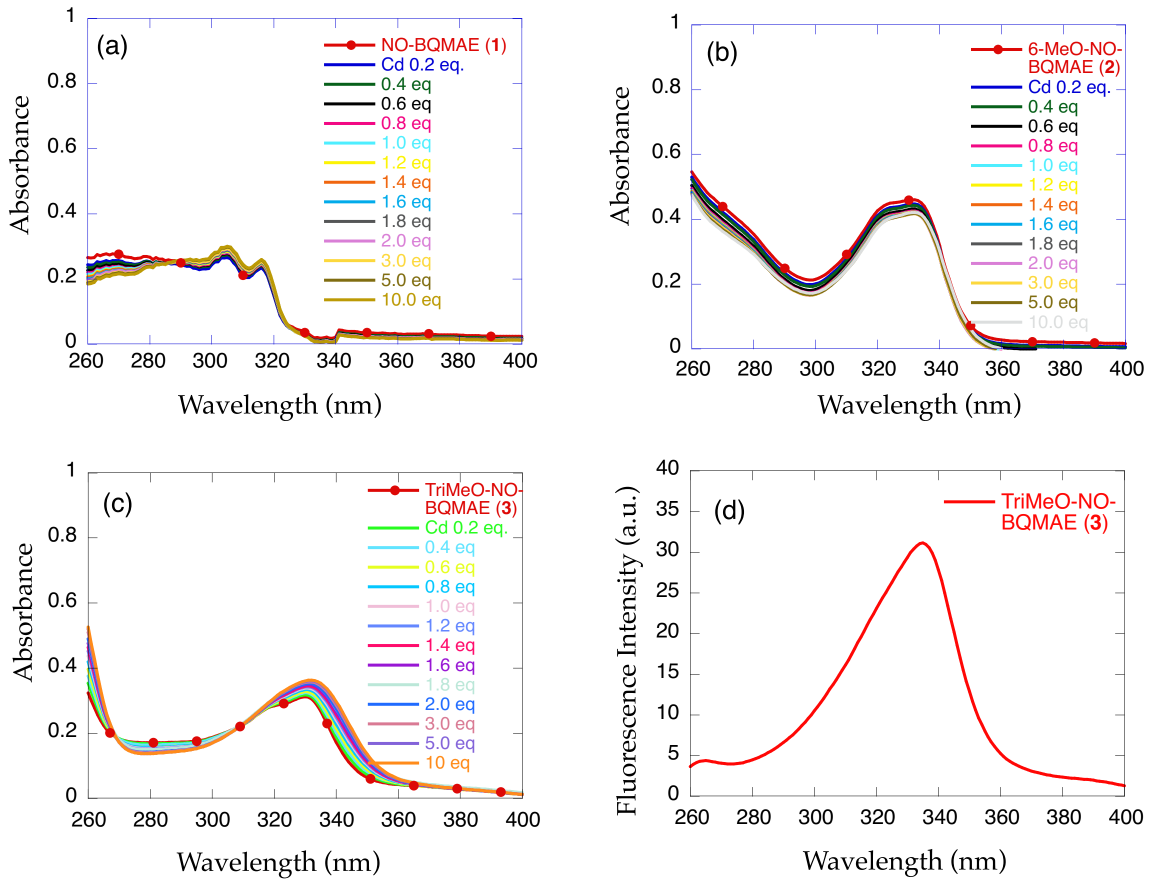

Figure S1. (a-c) Absorbance spectral changes of 34 μM (a) *N,O*-BQMAE (**1**), (b) 6-MeO-*N,O*-BQMAE (**2**), and (c) TriMeO-*N,O*-BQMAE (**3**) in DMF-HEPES buffer (1:1, 50 mM HEPES, 0.1 M KCl, pH = 7.5) at 25 °C in the presence of increasing concentrations of Cd^{2+}. (d) Excitation spectrum of 34 μM TriMeO-*N,O*-BQMAE (**3**) in DMF-HEPES buffer (1:1, 50 mM HEPES, 0.1 M KCl, pH = 7.5) at 25 °C (λ_{em} = 445 nm).

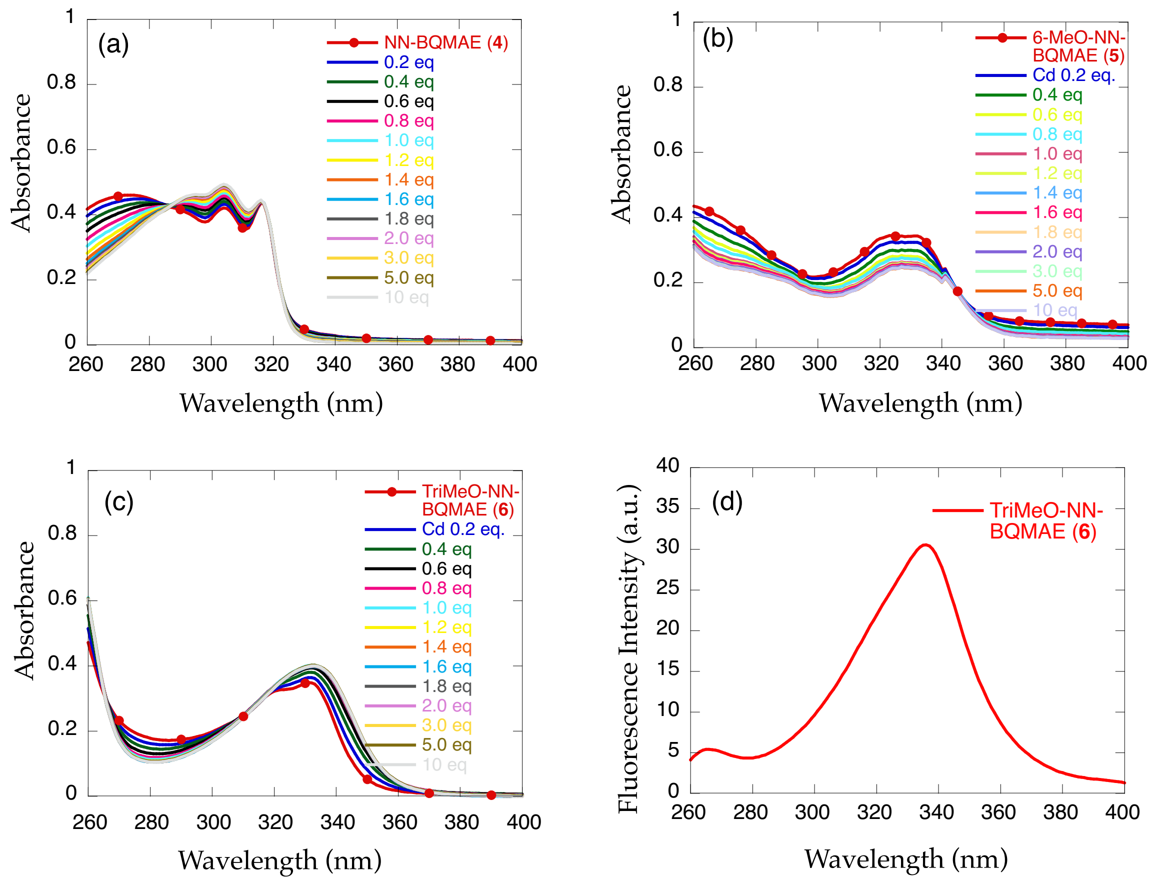

Figure S2. Absorbance spectral changes of 34 μM (a) *N*,*N*-BQMAE (**4**), (b) 6-MeO-*N*,*N*-BQMAE (**5**), and (c) TriMeO-*N*,*N*-BQMAE (**6**) in DMF-HEPES buffer (1:1, 50 mM HEPES, 0.1 M KCl, pH = 7.5) at 25 °C in the presence of increasing concentrations of Cd^{2+}. (d) Excitation spectrum of 34 μM TriMeO-*N*,*N*-BQMAE (**6**) in DMF-HEPES buffer (1:1, 50 mM HEPES, 0.1 M KCl, pH = 7.5) at 25 °C (λ_{em} = 450 nm).

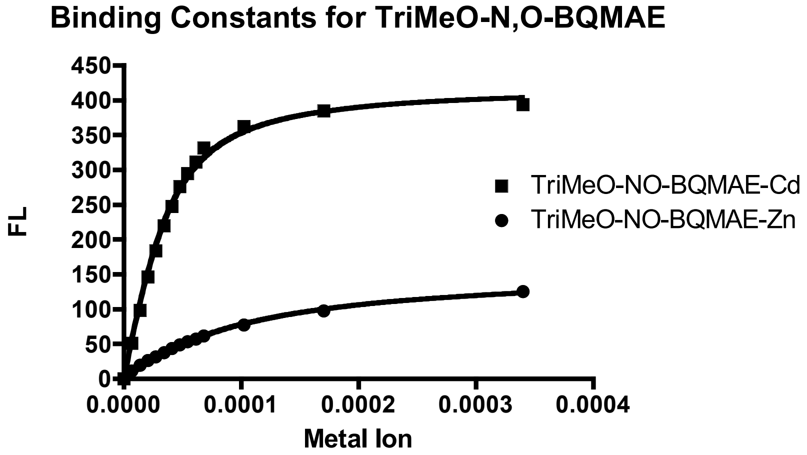

Metal ion	TriMeO-NO-BQMAE-Cd	TriMeO-NO-BQMAE-Zn
0.000000	0.0000	0.0000
6.800000e-06	51.1170	11.2370
1.360000e-05	98.3680	19.1750
2.040000e-05	146.4600	26.1420
2.720000e-05	183.8400	31.8570
3.400000e-05	219.6400	37.6700
4.080000e-05	247.3800	43.8500
4.760000e-05	276.0400	48.5700
0.000054	294.7900	53.4740
0.000061	311.6200	56.8450
0.000068	331.3400	61.6890
0.000102	362.4900	77.2350
0.000170	384.4300	97.3500
0.000340	393.6600	125.4100

	TriMeO-NO-BQMAE-Cd	TriMeO-NO-BQMAE-Zn
zinc binding 2/7/2008		
Best-fit values		
BMAX	422.3	155.7
KD	1.3942e-005	8.2378e-005
L0	3.4000e-005	3.4000e-005
Std. Error		
BMAX	7.325	3.254
KD	1.1612e-006	4.0810e-006
95% Confidence Intervals		
BMAX	406.4 to 438.3	148.6 to 162.8
KD	1.1412e-005 to 1.6472e-005	7.3486e-005 to 9.1271e-005
Goodness of Fit		
Degrees of Freedom	12	12
R squared	0.9964	0.9977
Absolute Sum of Squares	728.2	33.80
Sy.x	7.790	1.678
Constraints		
L0	L0 = 3.4000e-005	L0 = 3.4000e-005
Data		
Number of X values	14	14
Number of Y replicates	1	1
Total number of values	14	14
Number of missing values	0	0

Figure S3. Estimation of dissociation constants (K_d) for TriMeO-*N*,*O*-BQMAE (**3**) with Cd^{2+} and Zn^{2+} in DMF-HEPES buffer (1:1, 50 mM HEPES, 0.1 M KCl, pH = 7.5) at 25 °C.

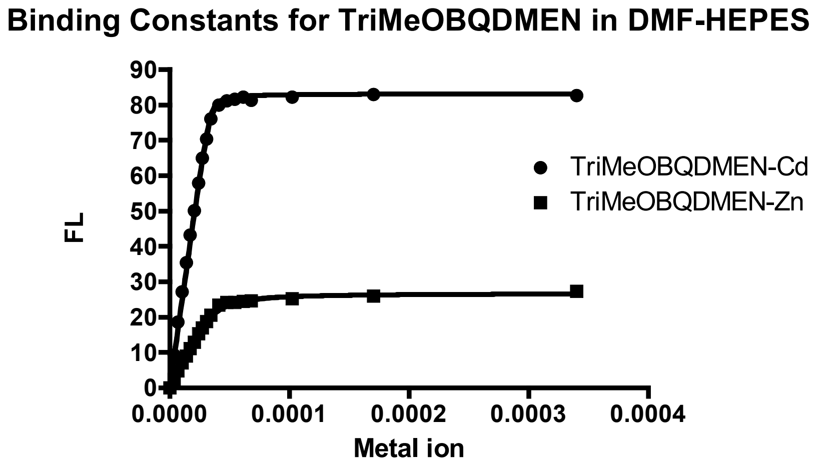

Metal ion	TriMeOBQDMEN-Cd	TriMeOBQDMEN-Zn
0.000000	0.0000	0.0000
3.400000e-06	9.3527	2.4409
6.800000e-06	18.5970	4.7305
1.020000e-05	27.1440	6.9822
1.360000e-05	35.3300	9.0172
1.700000e-05	43.2540	11.0700
2.040000e-05	50.1000	12.9260
2.380000e-05	57.9580	15.3450
2.720000e-05	64.8850	16.9910
3.060000e-05	70.4170	18.7420
3.400000e-05	75.9780	20.5390
4.080000e-05	79.9820	23.3890
4.760000e-05	81.1540	24.1120
0.000054	81.6550	24.2070
0.000061	82.2410	24.4270
0.000068	81.3670	24.5970
0.000102	82.2600	25.2590
0.000170	82.9060	25.9550
0.000340	82.6460	27.3420

	TriMeOBQDMEN-Cd	TriMeOBQDMEN-Zn
zinc binding 2/7/2008		
Best-fit values		
BMAX	83.20	26.78
KD	2.5822e-007	2.6953e-006
L0	3.4000e-005	3.4000e-005
Std. Error		
BMAX	0.6129	0.3782
KD	9.1225e-008	4.1199e-007
95% Confidence Intervals		
BMAX	81.90 to 84.49	25.98 to 27.58
KD	6.5736e-008 to 4.5070e-007	1.8260e-006 to 3.5646e-006
Goodness of Fit		
Degrees of Freedom	17	17
R squared	0.9979	0.9960
Absolute Sum of Squares	29.17	5.554
Sy.x	1.310	0.5716
Constraints		
L0	L0 = 3.4000e-005	L0 = 3.4000e-005
Data		
Number of X values	19	19
Number of Y replicates	1	1
Total number of values	19	19
Number of missing values	0	0

Figure S4. Estimation of dissociation constants (K_d) for TriMeOBQDMEN with Cd^{2+} and Zn^{2+} in DMF-HEPES buffer (1:1, 50 mM HEPES, 0.1 M KCl, pH = 7.5) at 25 °C.

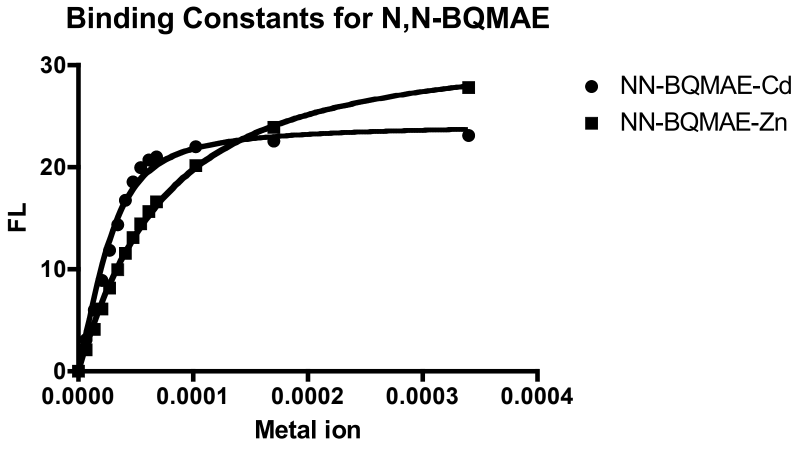

Metal ion	NN-BQMAE-Cd	NN-BQMAE-Zn
0.000000	0.0000	0.0000
6.800000e-06	3.1244	2.0859
1.360000e-05	6.0068	4.0858
2.040000e-05	8.9059	6.1217
2.720000e-05	11.8270	8.1550
3.400000e-05	14.3630	9.9381
4.080000e-05	16.7620	11.5270
4.760000e-05	18.5480	13.0970
0.000054	19.9370	14.4450
0.000061	20.6780	15.6370
0.000068	20.9850	16.6130
0.000102	22.0060	20.1280
0.000170	22.5630	23.9060
0.000340	23.1240	27.8210

	NN-BQMAE-Cd	NN-BQMAE-Zn
zinc binding 2/7/2008		
Best-fit values		
BMAX	24.34	32.60
KD	8.1238e-006	5.1613e-005
L0	3.4000e-005	3.4000e-005
Std. Error		
BMAX	0.7345	0.5430
KD	1.5398e-006	2.3897e-006
95% Confidence Intervals		
BMAX	22.74 to 25.94	31.42 to 33.79
KD	4.7686e-006 to 1.1479e-005	4.6405e-005 to 5.6820e-005
Goodness of Fit		
Degrees of Freedom	12	12
R squared	0.9878	0.9982
Absolute Sum of Squares	9.428	1.576
Sy.x	0.8864	0.3624
Constraints		
L0	L0 = 3.4000e-005	L0 = 3.4000e-005
Data		
Number of X values	14	14
Number of Y replicates	1	1
Total number of values	14	14
Number of missing values	0	0

Figure S5. Estimation of dissociation constants (K_d) for *N,N*-BQMAE (**4**) with Cd^{2+} and Zn^{2+} in DMF-HEPES buffer (1:1, 50 mM HEPES, 0.1 M KCl, pH = 7.5) at 25 °C.

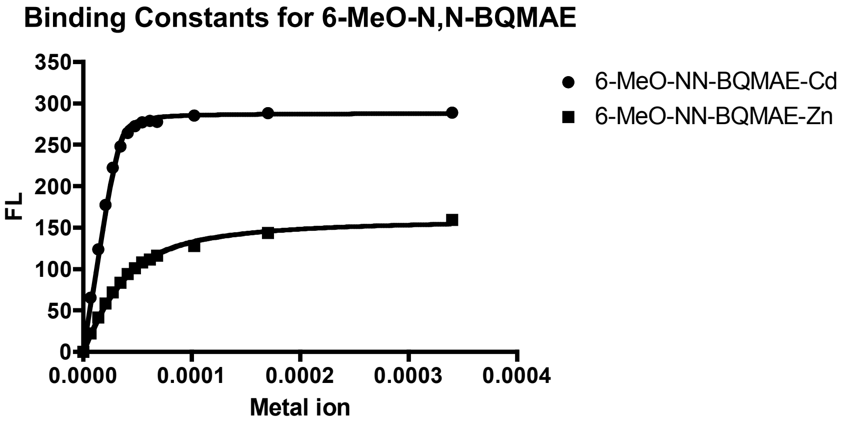

Metal ion	6-MeO-NN-BQMAE-Cd	6-MeO-NN-BQMAE-Zn
0.000000	0.0000	0.0000
6.800000e-06	65.3900	21.9210
1.360000e-05	123.9200	41.4690
2.040000e-05	177.2100	58.0740
2.720000e-05	222.5100	72.0130
3.400000e-05	248.1700	83.6360
4.080000e-05	264.0200	93.9660
4.760000e-05	272.5700	100.6500
0.000054	276.9800	107.7000
0.000061	279.0500	111.3900
0.000068	277.7400	115.8900
0.000102	285.0900	127.7000
0.000170	288.1100	143.4800
0.000340	288.8300	159.1300

	6-MeO-NN-BQMAE-Cd	6-MeO-NN-BQMAE-Zn
zinc binding 2/7/2008		
Best-fit values		
BMAX	288.2	162.4
KD	6.4235e-007	1.6384e-005
L0	3.4000e-005	3.4000e-005
Std. Error		
BMAX	3.578	2.524
KD	2.2847e-007	1.1333e-006
95% Confidence Intervals		
BMAX	280.4 to 295.9	156.9 to 167.9
KD	1.4451e-007 to 1.1402e-006	1.3915e-005 to 1.8853e-005
Goodness of Fit		
Degrees of Freedom	12	12
R squared	0.9954	0.9971
Absolute Sum of Squares	520.6	79.27
Sy.x	6.587	2.570
Constraints		
L0	L0 = 3.4000e-005	L0 = 3.4000e-005
Data		
Number of X values	14	14
Number of Y replicates	1	1
Total number of values	14	14
Number of missing values	0	0

Figure S6. Estimation of dissociation constants (K_d) for 6-MeO-*N,N*-BQMAE (**5**) with Cd^{2+} and Zn^{2+} in DMF-HEPES buffer (1:1, 50 mM HEPES, 0.1 M KCl, pH = 7.5) at 25 °C.

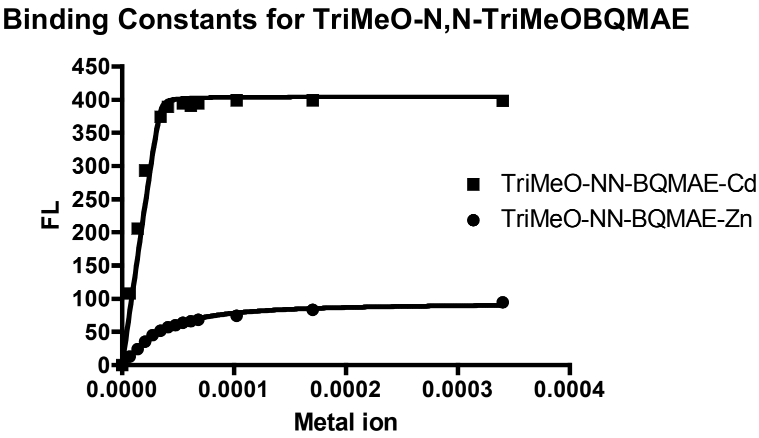

Metal ion	TriMeO-NN-BQMAE-Cd	TriMeO-NN-BQMAE-Zn
0.000000	0.0000	0.0000
0.000007	108.1000	12.4700
0.000014	205.7600	24.2960
0.000020	292.9800	35.6160
0.000027	~~353.3200~~	45.0430
0.000034	373.9900	51.4780
0.000041	389.1300	56.7180
0.000048	~~393.0700~~	60.3440
0.000054	394.5300	63.8650
0.000061	390.8200	65.9310
0.000068	394.7800	67.9110
0.000102	399.1800	74.3840
0.000170	399.0200	83.3340
0.000340	398.3100	94.6870

	TriMeO-NN-BQMAE-Cd	TriMeO-NN-BQMAE-Zn
zinc binding 2/7/2008		
Best-fit values		
BMAX	404.4	94.26
KD	1.3580e-007	1.4635e-005
L0	3.4000e-005	3.4000e-005
Std. Error		
BMAX	11.16	2.273
KD	3.0388e-007	1.6558e-006
95% Confidence Intervals		
BMAX	379.5 to 429.2	89.31 to 99.22
KD	-5.4123e-007 to 8.1284e-007	1.1027e-005 to 1.8243e-005
Goodness of Fit		
Degrees of Freedom	10	12
R squared	0.9704	0.9927
Absolute Sum of Squares	5997	68.35
Sy.x	24.49	2.387
Constraints		
L0	L0 = 3.4000e-005	L0 = 3.4000e-005
Data		
Number of X values	14	14
Number of Y replicates	1	1
Total number of values	12	14
Number of missing values	2	0

Figure S7. Estimation of dissociation constants (K_d) for TriMeO-*N,N*-BQMAE (**6**) with Cd^{2+} and Zn^{2+} in DMF-HEPES buffer (1:1, 50 mM HEPES, 0.1 M KCl, pH = 7.5) at 25 °C.

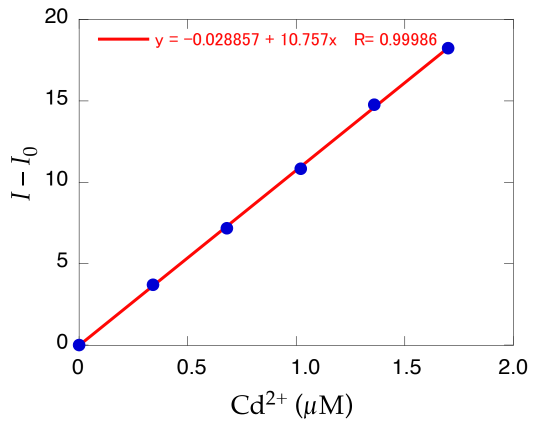

Figure S8. Estimation of LOD (limit of detection) for Cd^{2+} with TriMeO-*N*,*N*-BQMAE (**6**) in DMF-HEPES buffer (1:1, 50 mM HEPES, 0.1 M KCl, pH = 7.5) at 25 °C. The 3σ value (σ corresponds to standard deviation from 6 measurements) of blank solution (34 μM TriMeOBQMOA (**9**)) is 0.183402 in fluorescence intensity unit, which corresponds to 17 nM from the slope of the liner dynamic fluorescence intensity plot (*k*) shown above (LOD = 3σ/*k*). The LOQ (limit of quantitation) is calculated to be 57 nM (LOQ = 10σ/*k*).

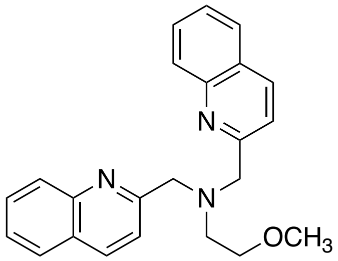

N,N-BQMAE (**4**)

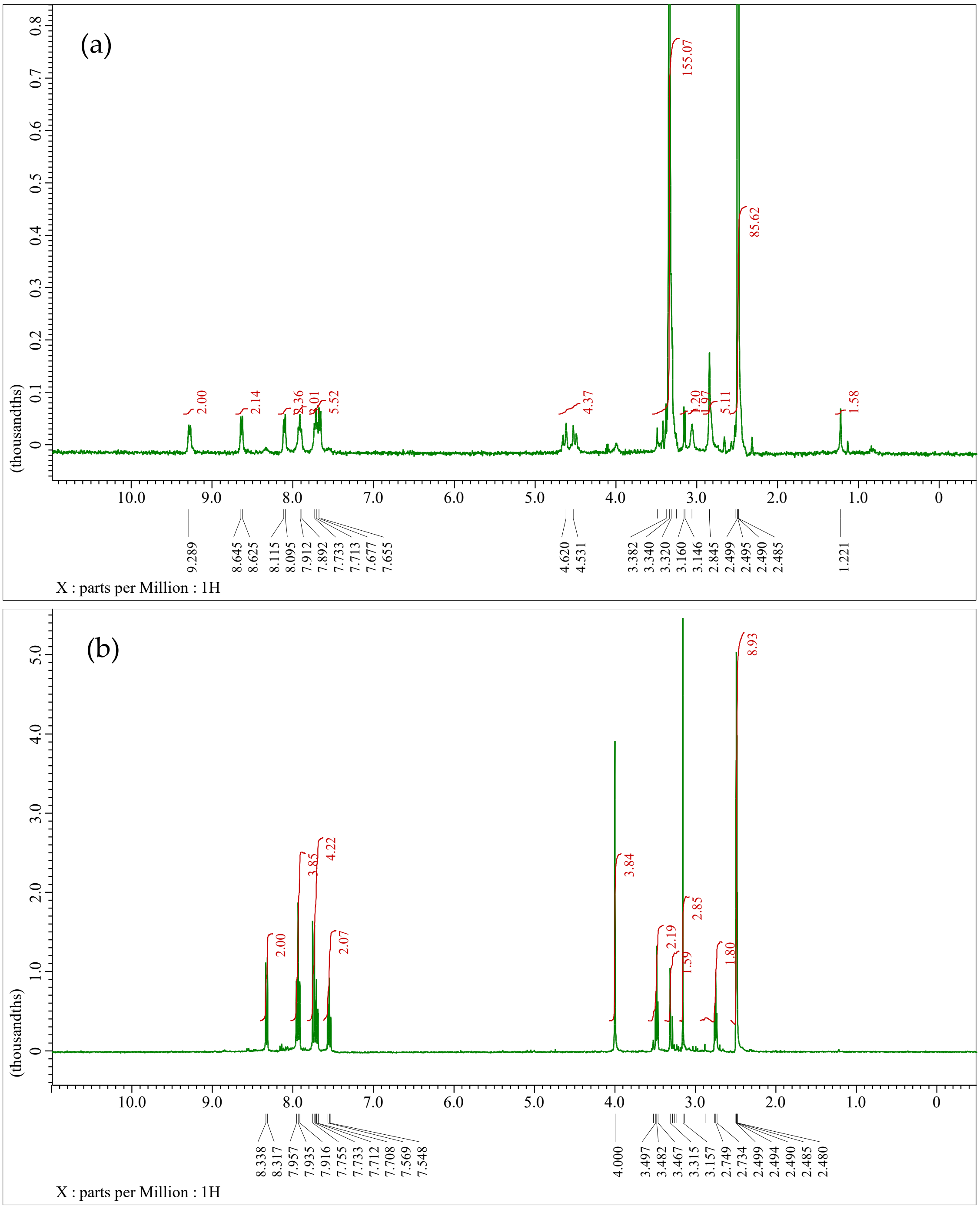

Figure S9. ^{1}H NMR spectra of (a) Cd^{2+} complex and (b) free ligand of *N,N*-BQMAE (**4**) in DMSO-d_6.

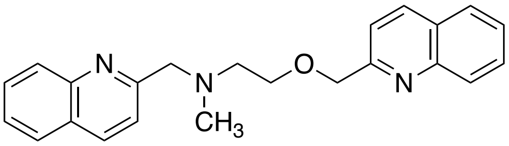

N,O-BQMAE (**1**)

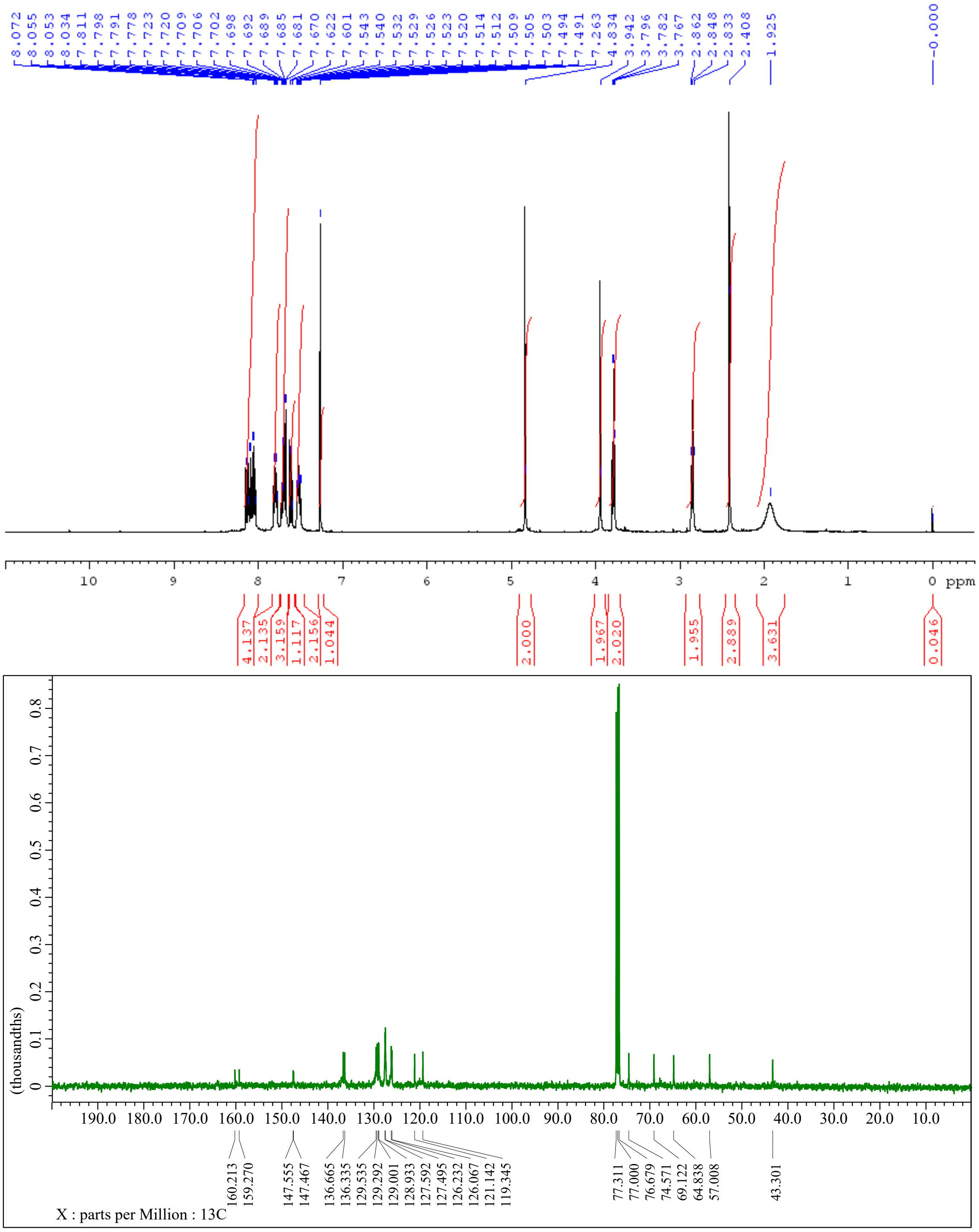

Figure S10. $^{1}H/^{13}C$ NMR spectrum of *N,O*-BQMAE (**1**) in $CDCl_3$.

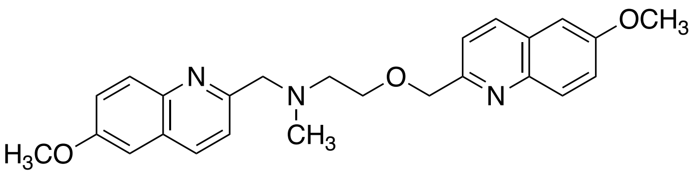

6-MeO-*N,O*-BQMAE (**2**)

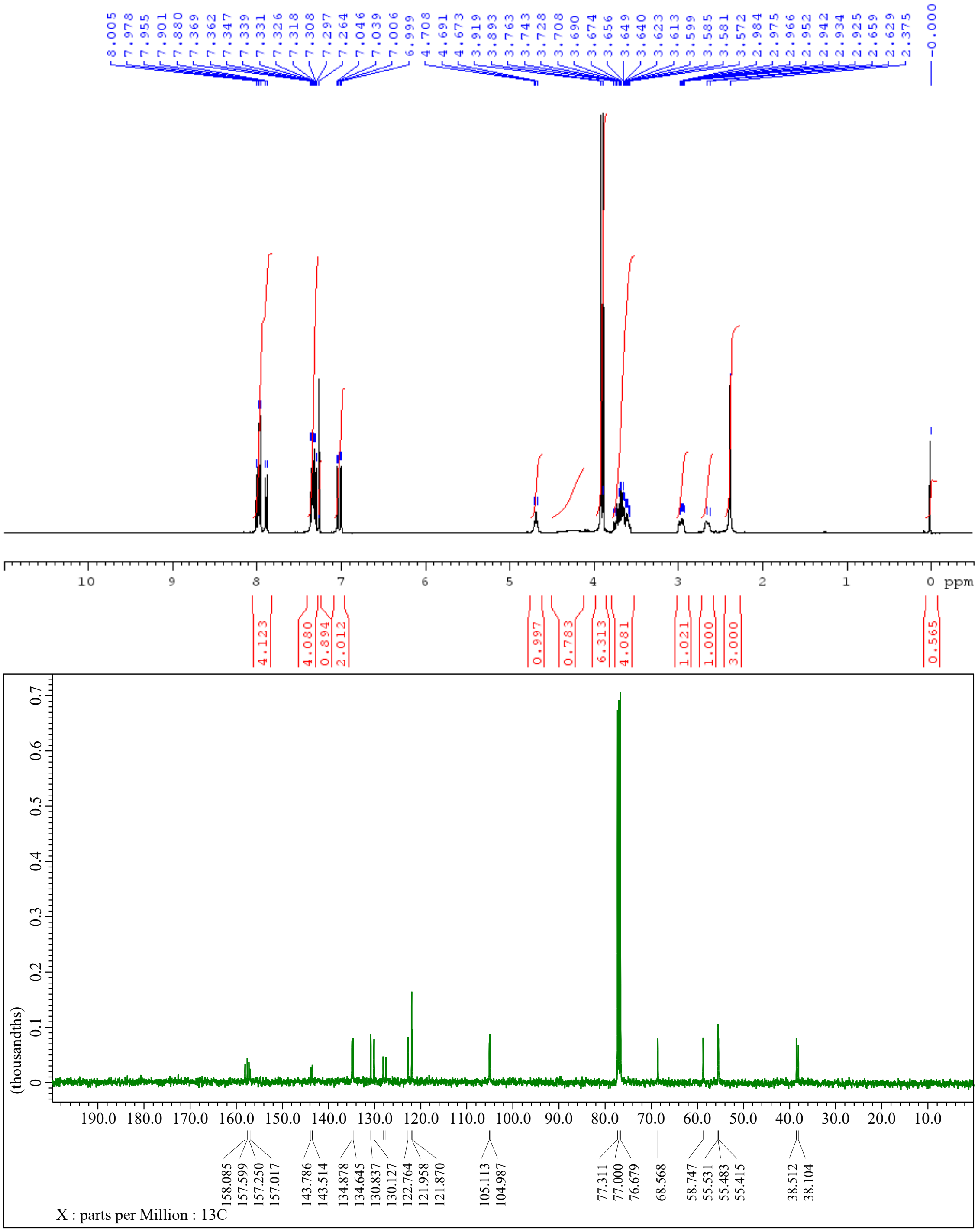

Figure S11. ^{1}H/^{13}C NMR spectrum of 6-MeO-*N,O*-BQMAE (**2**) in $CDCl_3$.

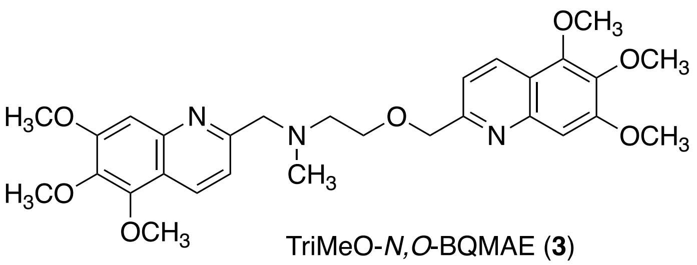

TriMeO-*N*,*O*-BQMAE (**3**)

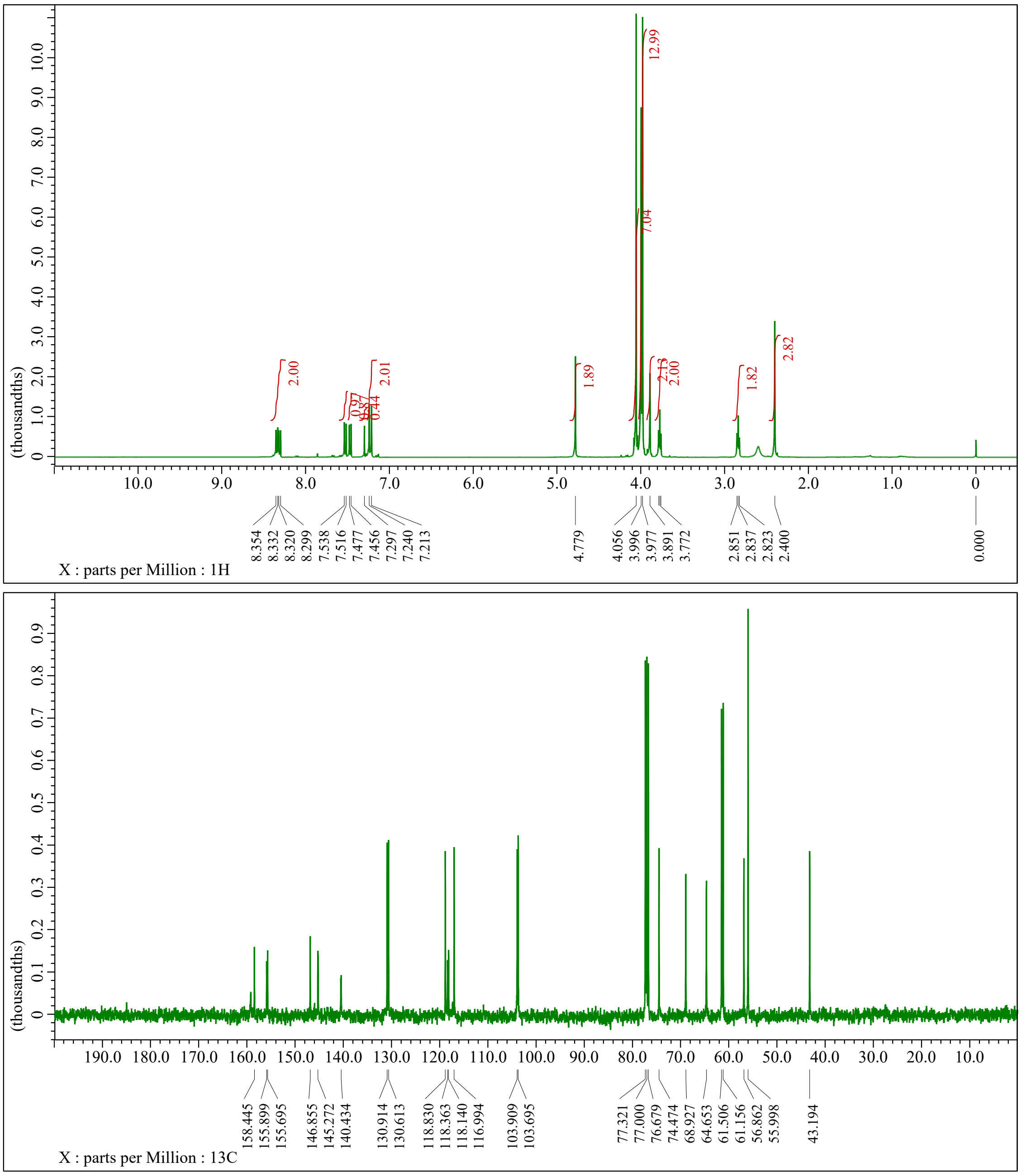

Figure S12. $^{1}H/^{13}C$ NMR spectrum of TriMeO-*N*,*O*-BQMAE (**3**) in $CDCl_3$.

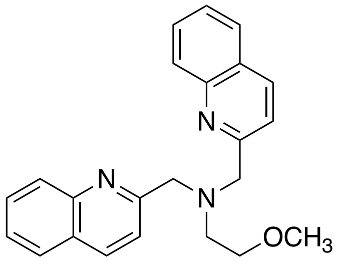

N,N-BQMAE (**4**)

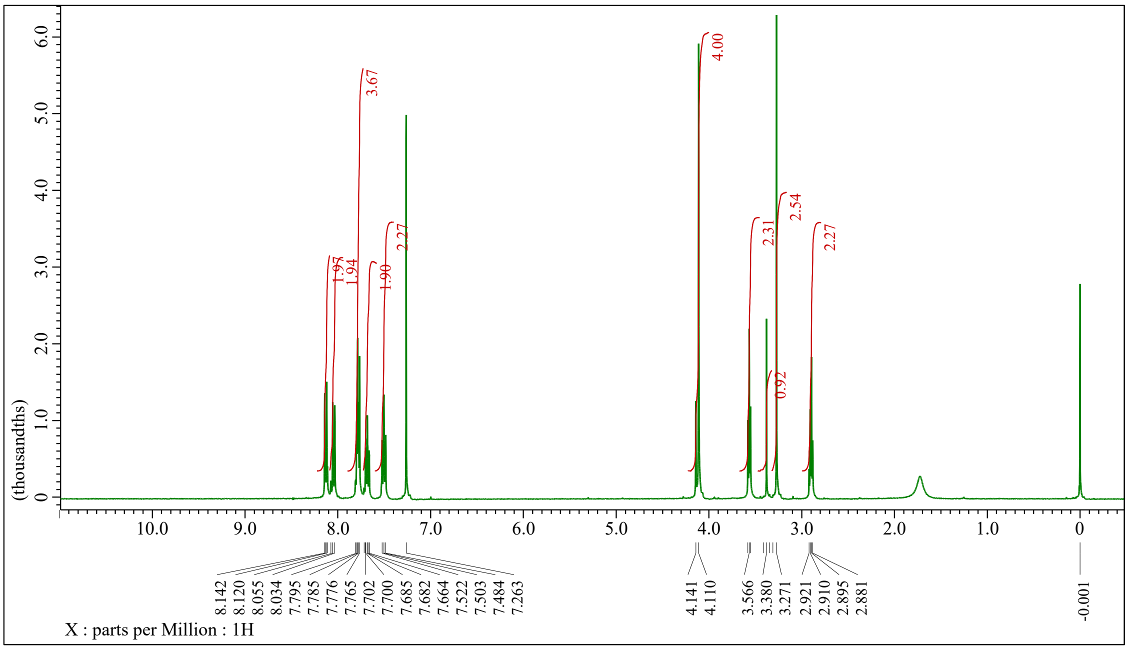

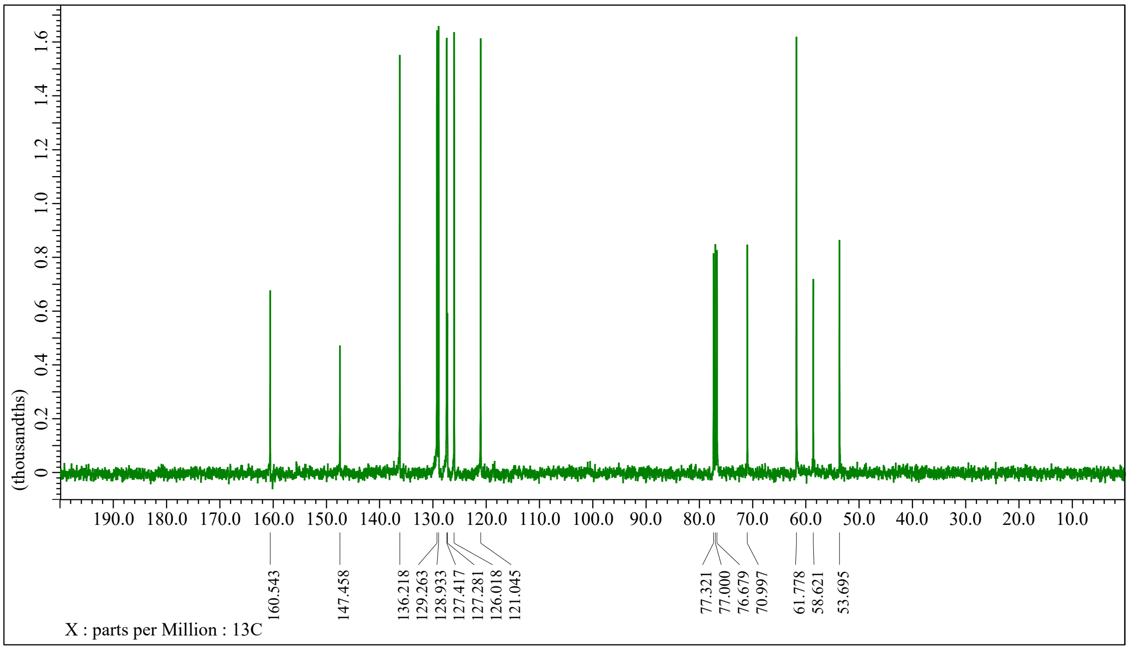

Figure S13. $^{1}H/^{13}C$ NMR spectrum of *N,N*-BQMAE (**4**) in $CDCl_3$.

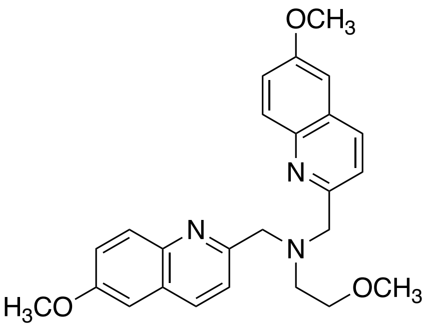

6-MeO-*N,N*-BQMAE (**5**)

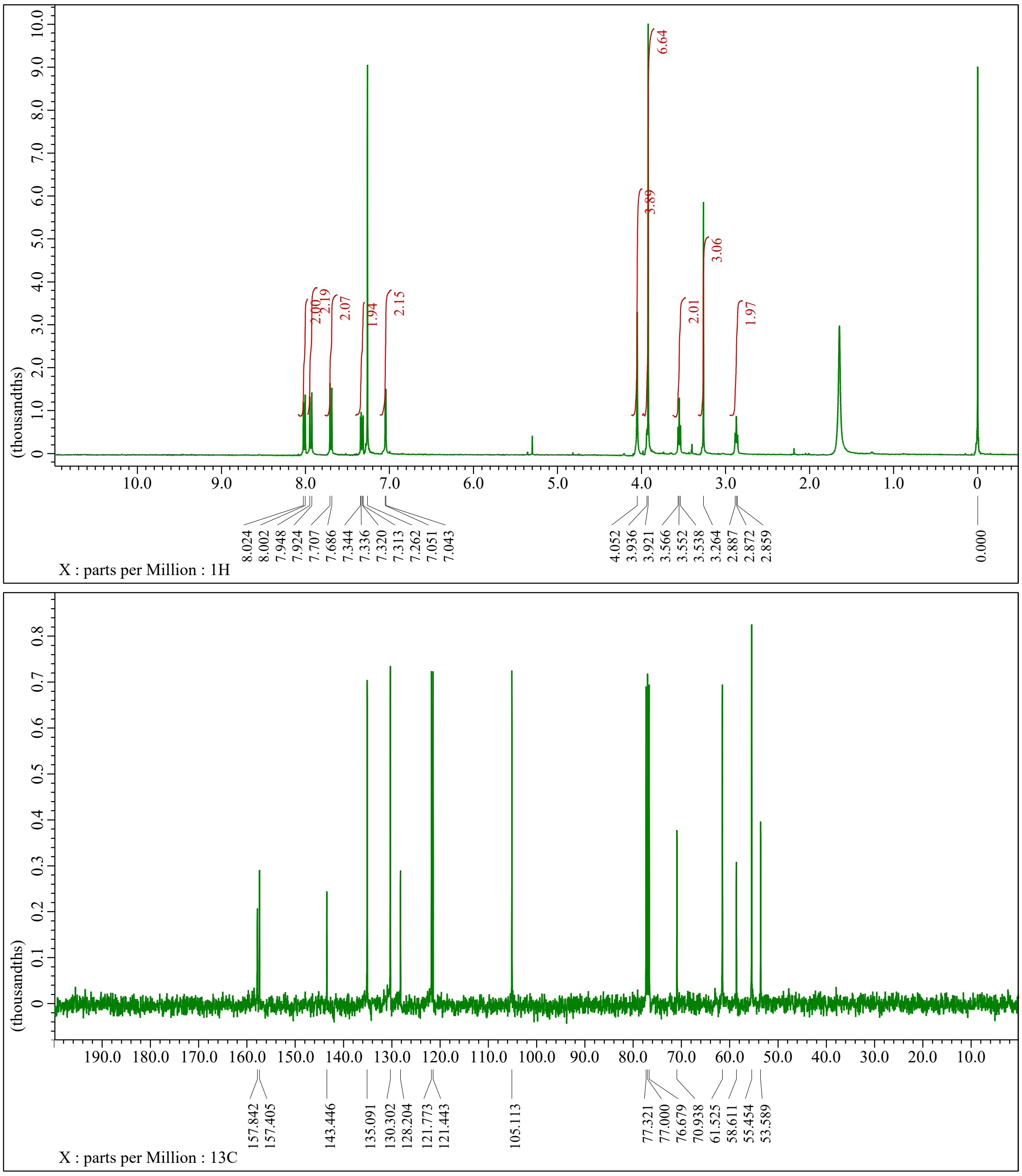

Figure S14. $^{1}H/^{13}C$ NMR spectrum of 6-MeO-*N,N*-BQMAE (**5**) in $CDCl_3$.

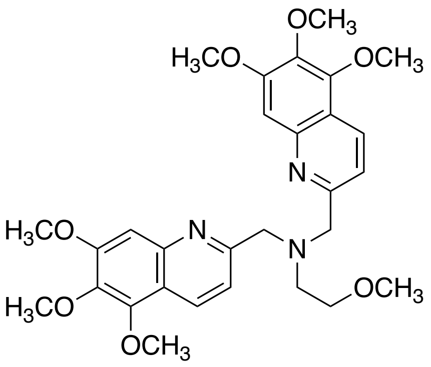

TriMeO-*N*,*N*-BQMAE (**6**)

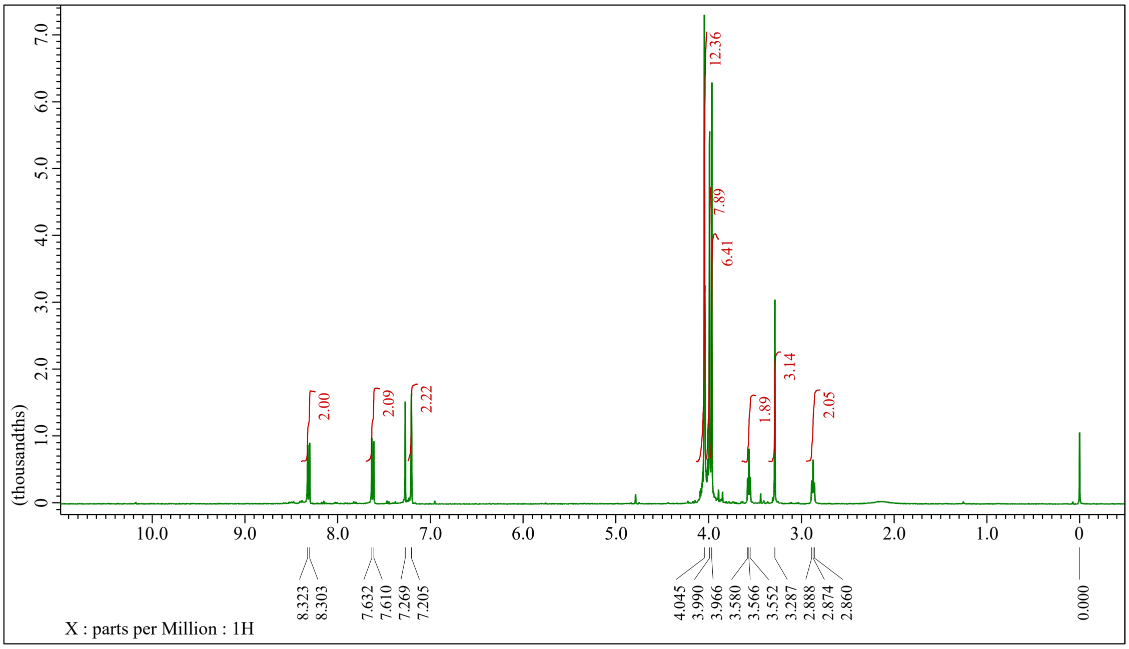

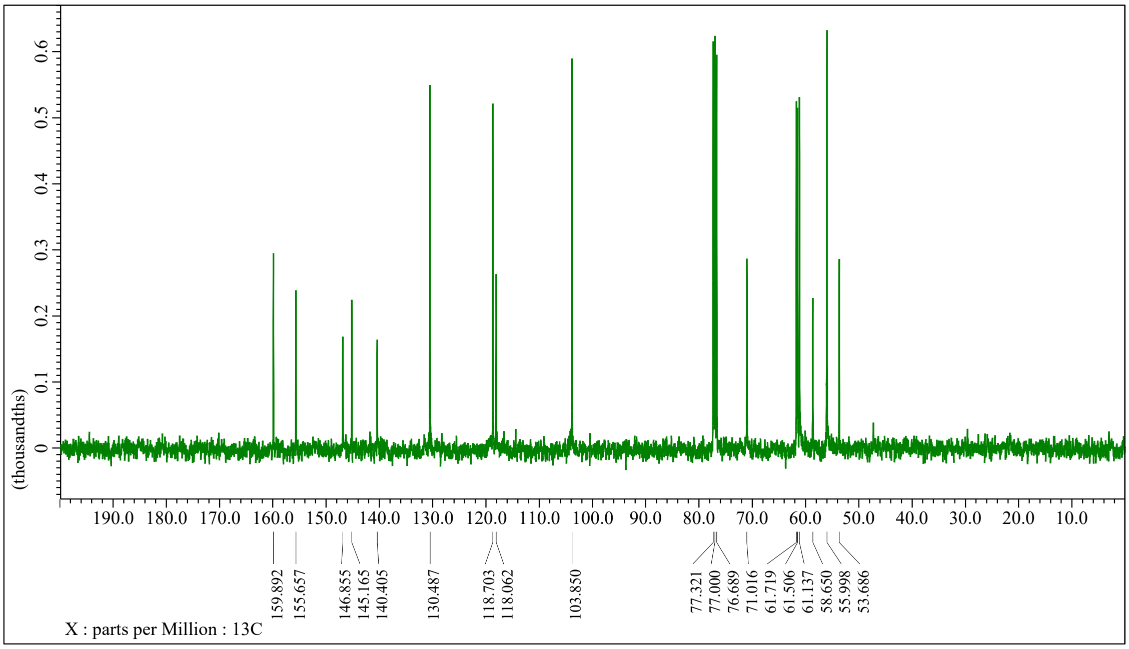

Figure S15. $^{1}H/^{13}C$ NMR spectrum of TriMeO-*N*,*N*-BQMAE (**6**) in $CDCl_3$.
